# Drivers of Intraspecific Trait Variation and Drought Response of a Dominant US Great Plains Grass: Disentangling the Role of Climate and Genetic Background

**DOI:** 10.1002/ece3.73584

**Published:** 2026-04-29

**Authors:** Jack Sytsma, Helen Winters, Matthew Galliart, Ryann Patterson, Brian Maricle, Loretta Johnson

**Affiliations:** ^1^ Division of Biology Kansas State University Manhattan Kansas USA; ^2^ Department of Biological Sciences Fort Hays State University Hays Kansas USA; ^3^ Department of Biology Saginaw Valley State University University Center Michigan USA

**Keywords:** *Andropogon gerardii*, C_4_ grass, environmental gradients, functional trait, grasslands, greenhouse, phenotypic plasticity

## Abstract

Intraspecific trait variation (ITV) is critical for plant adaptation, particularly under increasing drought. ITV arises from phenotypic plasticity and genetic differentiation, so identifying its sources improves understanding of population responses to drought. This study investigates how climate and genetics shape ITV and drought responses in *Andropogon gerardii* , a foundational Great Plains grass. To quantify climatic determinants of ITV, we conducted a common‐garden greenhouse experiment with 25 *A. gerardii* populations spanning wide temperature (4°C–21°C; Minnesota–Texas) and precipitation (350–1400 mm year^−1^; Colorado–North Carolina) gradients, measuring 17 functional traits. Populations were also genotyped to assess the genetic basis of ITV, and greenhouse–field trait comparisons were used to separate genetic and environmental effects. To test drought as a selective pressure, eight populations from a precipitation gradient (470–1350 mm year^−1^) were exposed to experimental drought (15% moisture vs. 30% control). We hypothesized that climate of origin, particularly precipitation, predicts ITV: dry‐origin populations would express conservative traits (higher water‐use efficiency, shorter stature), whereas wet‐origin populations would exhibit competitive traits (greater height, biomass). We further predicted that drought would reduce growth and delay flowering, with stronger effects in wet‐origin populations, and that trait variation would correspond with genetic differentiation. Precipitation was the strongest predictor of ITV. Principal component analyses revealed that wet‐origin populations displayed competitive traits, while arid populations showed conservative traits. Congruent genetic and trait PCAs confirmed a genetic basis for ITV, and consistent greenhouse–field patterns reinforced genetic control. Under drought, wet‐origin populations exhibited greater declines in height and photosynthesis, indicating drought as a key selective force. Overall, ITV in *A. gerardii* is primarily shaped by precipitation through genetically based trait responses, offering a predictive framework for grassland adaptation under future climate change.

## Graphical abstract

This study explores how climate and genetics drive intraspecific trait variation (ITV) and drought responses in *Andropogon gerardi* , a dominant grass shaping North American prairies. We found that precipitation strongly predicts genetically based trait differentiation, with dry‐origin populations showing drought resilience and wet‐origin populations favoring growth and competition. These results reveal how climate imprints adaptive genetic variation within species, providing a powerful framework for predicting ecosystem resilience and guiding restoration and conservation strategies under accelerating climate change.

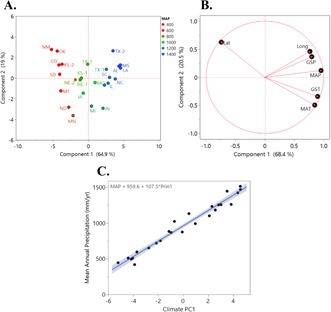

## 1 Introduction

Trait variation is a cornerstone of ecological and evolutionary processes, influencing how organisms acquire resources, tolerate stress, and interact with their environment (McGill et al. 2006; Violle et al. 2007; Laughlin et al. 2020). Intraspecific trait variation (ITV)—variation within species—is increasingly being recognized as equally important (Bolnick et al. 2011; Des Roches et al. 2018, 2021; Westerband, Funk, and Barton 2021) as interspecific variation. Understanding why ITV varies among populations, and whether it is driven primarily by phenotypic plasticity or genetic differentiation, remains a central challenge in ecology and evolution. For example, ITV helps explain how populations adapt to environmental change (Malyshev et al. 2016), accounting for up to 25% of trait variation within communities (Siefert et al. 2015). Plant ITV is a powerful driver of ecosystem dynamics—at times surpassing interspecific differences in shaping communities and ecosystem functions (Des Roches et al. 2018). However, the magnitude of ITV often varies across environmental gradients, particularly climatic gradients (Westerband, Knight, and Barton 2021). This raises the question of what mechanisms generate this variation across broad environmental gradients, and fuels a growing recognition of the role of ITV in shaping species' responses to climate (Valladares et al. 2014).

A key source of ITV is phenotypic plasticity—the ability of a genotype to express different trait values under different environmental conditions—while another is genetic differentiation among populations (Sork 2018) shaped by historical selection. Disentangling these mechanisms is critical for predicting how species will respond to ongoing climate change. Common gardens, where all experimental plants share the same conditions, are foundational tools for disentangling sources of ITV (Turesson 1922; VanWallendael et al. 2022; Schwinning et al. 2022; Johnson et al. 2022). This approach is particularly powerful in the context of climate gradients: it allows researchers to test whether populations from contrasting environments consistently exhibit differences in functional traits and whether those differences align with their climate‐of‐origin (Schwinning et al. 2022).

In contrast to controlled common garden studies, field studies capture trait variation under ecologically realistic conditions, integrating the effects of local climate, soil, and species interactions (Breitschwerdt et al. 2019; Westerband, Funk, and Barton 2021). These approaches are complementary: common gardens offer mechanistic insight and experimental control, while field observations provide ecological realism and context (Sork 2018). Together, they allow researchers to ask whether population‐level trait differences observed in nature persist under common conditions, or instead reflect plastic responses to local environments.

Combining both field and common garden approaches is especially powerful, as it clarifies not only how traits vary, but which sources of variation—genetic, environmental, or their interaction (Groot et al. 2018)—are driving observed patterns across a species' range. By including genomic data, researchers can directly test whether trait differences among populations reflect underlying genetic divergence rather than site‐level physiological adjustment to environment (Schwinning et al. 2022; Johnson et al. 2022). Integrating trait, genetic, and environmental data therefore provides a mechanistic framework for understanding how ITV emerges and how it may shape future responses to climate stressors such as drought.

When paired with targeted environmental manipulations (e.g., water availability), common gardens can further reveal whether climate‐of‐origin predicts trait values and whether populations differ in their responses to stress under standardized conditions (Schwinning et al. 2022; Johnson et al. 2022). Such experiments directly address whether some populations are inherently more drought tolerant—expressing traits such as shorter stature associated with drought conditions—or whether observed responses primarily reflect short‐term plasticity. Previous field measurements of the same populations used in this experiment (Sytsma et al. 2026) revealed regional trait shifts, including potential drought‐associated short‐stature phenotypes. Identifying whether populations differ in drought tolerance, and whether this variation corresponds to climate‐of‐origin, is critical for anticipating species' responses to increasing water limitation under predicted droughts (Gazol et al. 2023).

Here, our overarching goal is to determine how phenotypic plasticity and genetic differentiation contribute to morphological and physiological ITV across a broad climate gradient, and how this variation influences population responses to drought. We address this question using *Andropogon gerardii* Vitman (big bluestem), a dominant C_4_ perennial grass and foundation species of the US Great Plains (Rogler 1944; Knapp et al. 1998). We examined 25 populations spanning over 2000 km across the species' range, encompassing wide climatic gradients in mean temperature (4°C–21°C; Minnesota to Texas, USA) and precipitation (350–1400 mm year^−1^; Colorado to North Carolina).

To test whether these patterns reflect genetic differentiation or environmental acclimation, we collected seed from the same populations and grew them under controlled greenhouse conditions, enabling direct comparisons of field and greenhouse trait variation. If populations from dry regions remain short‐statured under well‐watered conditions, this would suggest a genetic basis of the trait (Ramírez‐Valiente et al. 2022); if plants increase growth when water limitation is removed, this would indicate strong environmental control and plasticity (Martínez‐Sancho et al. 2025). While *A. gerardii* productivity declines along the east–west rainfall gradient of the US Great Plains (Weaver 1954; Sala et al. 1988; Epstein et al. 1998), the mechanisms underlying this pattern—plastic physiological limitation versus genetically constrained conservative phenotypes—remain unresolved (Gupta et al. 2020; Juenger 2013).

To address how ITV in *A. gerardii* reflects climate adaptation and genetic differentiation, we pursued four integrated objectives. First, in objective 1, we quantified the magnitude and structure of ITV across populations in a greenhouse common garden to test whether trait variation aligns with climate‐of‐origin. We hypothesized that populations from more arid environments would express drought‐tolerant, conservative trait syndromes, whereas populations from wetter climates would exhibit more acquisitive traits. In objective 2, we assessed whether ITV reflects underlying genetic differentiation using genotyping by sequencing, predicting that genetic structure would correlate with trait variation among populations. In objective 3, we compared ITV patterns between greenhouse and field environments to test whether population‐level trait differences are retained across environments, as expected if traits are under genetic control. Finally, in objective 4, we conducted a controlled drought experiment to evaluate whether populations differ in drought sensitivity, hypothesizing that populations from wetter regions would show stronger declines in their performance responses to drought. By integrating common garden experiments, genomic data, field observations, and drought manipulations across a large portion of the species' range, this study directly addresses the longstanding challenge of disentangling genetic differentiation from phenotypic plasticity in shaping ITV.

## 2 Materials and Methods

### 2.1 Seed Collection and Germination

**FIGURE 1:**
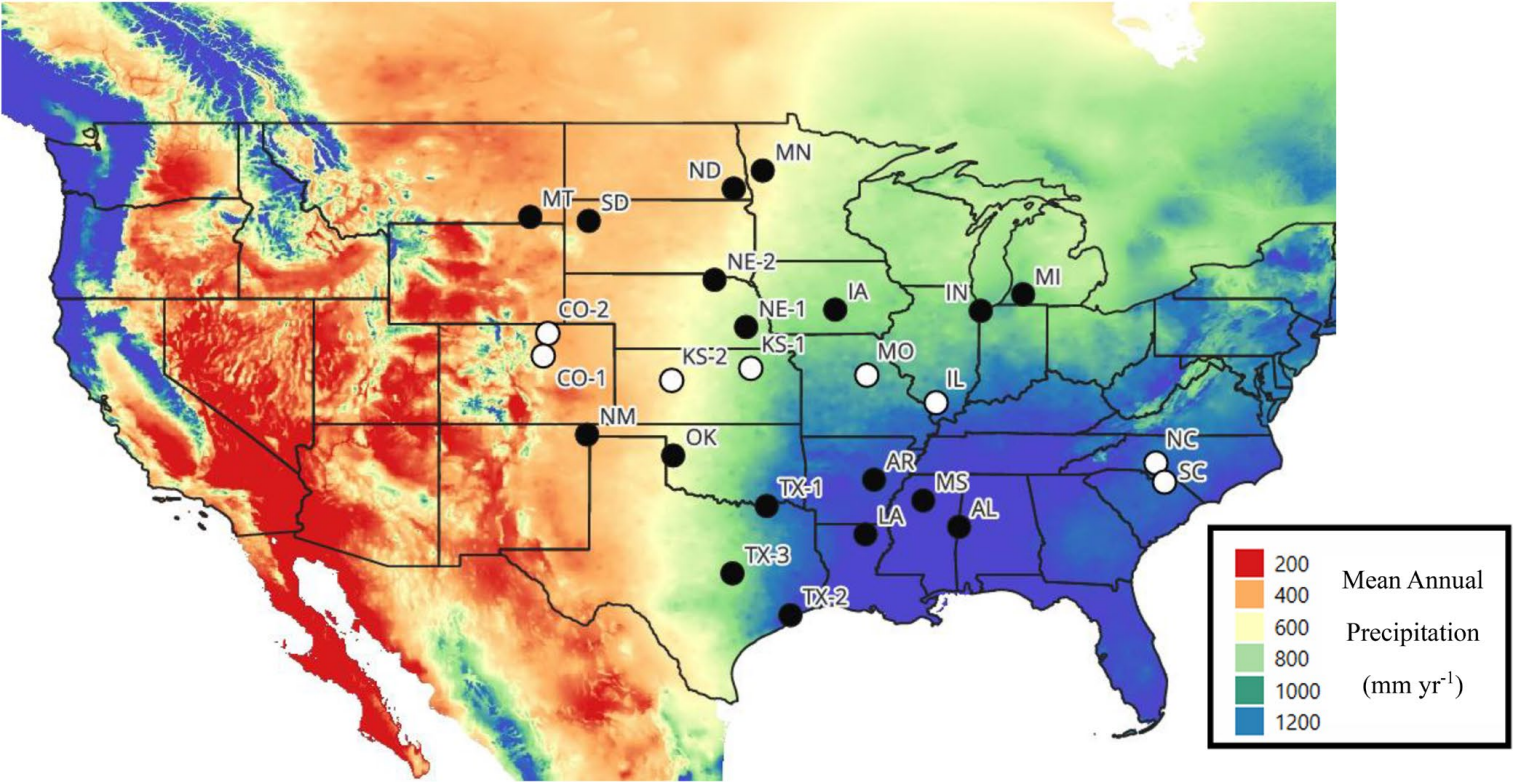
Map of Sites *A. gerardii*. A. from 25 source populations across a precipitation gradient. Points indicate all 25 source populations and hollow points indicate source populations for the drought experiment of eight populations.

Our main experiment (Obj. 1) included all populations sourced from gradients of MAT 4°C–21°C and 350–1400 mm year^−1^ MAP (Figure 1A). Seed of 25 *A. gerardii* populations were collected by hand Sept‐Dec 2023 from sites located across the range of this species (Figure 1). These seed were collected at the same locations as the field populations studied in Sytsma et al. (2026), but not from the same exact plants. Seeds were scarified by gently rubbing them with a rubber abrasive by hand until the seed coat was visibly worn and then sown into germination trays containing Berger BM2 germination blend potting soil in late February 2024. The germination medium was composed primarily of peat moss, perlite, vermiculite, and limestone, with a low fertilizer starter (15 mg N, 10 mg P, and 15 mg K per liter of soil) to support early seedling growth. Soil moisture and nutrient conditions were consistent across all pots. Trays were watered to maintain surface moisture until seedlings reached a sufficient size for transplanting. Then seedlings were transplanted into pots (20 × 10 × 10 cm) containing Berger BM6 general purpose soil mix.

#### 2.1.1 Source Population Climate Data

We downloaded mean (30‐year normal) climatic variable from the ClimateNA version 7.3 database based on GPS coordinates where *A. gerardii* seed was collected. The main experiment source populations ranged from 418 to 1413 mm year^−1^ historic (30 year averages, 1990–2020) mean annual precipitation (MAP; Figure 1A), 5.8°C–21.2°C historic mean annual temperature (MAT; Table S1). The source populations from the drought experiment ranged 472–1356 mm year^−1^ MAP (Figure 1B). The full climatic dataset is found in Table S1.

#### 2.1.2 Experimental Design: Greenhouse Common Garden Study

Each population (25) had six replicate plants (6 × 25 = 150 total plants) that were watered daily to maintain field capacity (~30% soil moisture, mean = 31.4%; measured with ECH_2_0 10HS Sensors; Meter Group, Pullman, WA, USA; Figure S1). Plants were randomized in a block design across greenhouse benches, where each block consisted of a group of plants on the same bench to account for potential spatial variation in light or temperature. Watering was applied manually to each individual plant rather than at the block level, ensuring that soil moisture targets were maintained independently for each pot.

Volumetric soil water content (m^3^ m^−3^) was measured daily using METER soil moisture probes (ECH_2_0 10HS Onset S‐SMD‐M005 Large Volume Soil Moisture Sensor, 15 cm depth, pot depth 20 cm) to monitor soil moisture. One reading was measured per block (six readings per day; Figure S1) as well as all plants in the experiment (*n* = 150) weekly.

We maintained the greenhouse at ~25°C (range: 24°C–28°C) with no supplemental lighting. The greenhouse was in Manhattan, Kansas, USA (39.2° N, 96.5° W); the experiment ran from May 15 to August 25, 2025. Ambient light levels in the greenhouse remained between 900 and 1200 μmol m^−**2**^ s^−**1**^ between 10:00 and 14:00 throughout the course of both greenhouse experiments.

#### 2.1.3 Functional Traits

To quantify ITV in relation to climate‐of‐origin, we conducted measurements of 17 morphological, physiological, and phenological traits in *A. gerardii* (Table 1).

**TABLE 1:** Summary of functional traits measured in the main greenhouse experiment and drought experiment.

Trait category	Trait	Description	Main experiment (25 pops, *n* = 6 plants per pop)	Drought experiment (8 pops, *n* = 8 plants per pop/treatment)
Morphological	Vegetative height	Maximum extended leaf height (cm), excluding reproductive stalk	Measured weekly, analyzed as seasonal mean	Measured weekly, analyzed Day 38 post‐drought
Morphological	Leaf width	Mean width of three mature leaves (mm)	As above	As above
Morphological	Leaf thickness	Mean thickness of three mature leaves, excluding midvein (mm)	Measured 3×; analyzed as seasonal mean	Measured 3×; analyzed day 38 post‐drought
Morphological	Leaf area	Projected area from digital images (cm^2^)	As above	As above
Morphological	Number of leaves	Total leaves per plant	Measured at end of experiment	Measured at end of experiment
Morphological	Reproductive stalk diameter	Diameter measured 15 cm above soil surface (mm)	As above	As above
Morphological	Aboveground biomass	Total aboveground biomass	Not measured	Measured at harvest
Morphological	Belowground Biomass	Total belowground biomass	Not measured	As above
Morphological	Above: belowground biomass	Total aboveground biomass/total belowground biomass	Not measured	As above
Growth	Growth rate (height)	Change in height over time (cm cm^−1^ day^−1^)	Calculated from repeated measures	Calculated from repeated measures
Growth	Growth rate (leaf area)	Change in leaf area over time (cm^2^ cm^−2^ day^−1^)	As above	As above
Physiological	Photosynthetic rate	Net CO₂ assimilation, A (μmol CO₂ m^−2^ s^−1^)	Measured 3×; analyzed as seasonal mean	Measured 3×; analyzed day 38 post‐drought
Physiological	Stomatal conductance	gs (mol H_2_O m^−2^ s^−1^)	As above	As above
Physiological	Transpiration rate	E (mmol H_2_O m^−2^ s^−1^)	As above	As above
Physiological	Water‐use efficiency (WUE)	A/E	Measured 3×; analyzed as seasonal mean	Measured 3×; analyzed day 38 post‐drought
Physiological	Internal CO_2_ concentration	Ci (ppm)	As above	As above
Physiological	Chlorophyll absorbance	SPAD units (mean of ≥ 3 readings)	Weekly; analyzed as seasonal mean	Weekly; analyzed day 38 post‐drought
Physiological	Midday leaf water potential	Ψleaf (MPa)	Measured 3×; analyzed as seasonal mean	Measured 3×; analyzed day 38 post‐drought
Phenological	Date of bolting	Day of first flowering stalk	Monitored daily	Monitored daily
Phenological	Date of flowering	Day of first anther emergence	As above	As above

#### 2.1.4 Morphological Traits

To assess differences in plant morphology (Table 1), we measured vegetative height, leaf width, leaf thickness, leaf area, number of leaves, and reproductive stalk diameter. For height, we extended the longest leaf vertically and recorded to the nearest cm, excluding reproductive stalks. We measured leaf width by averaging three mature leaves at their broadest point. We assessed leaf thickness with a Mitutoyo hand caliper of an average of three leaves per plant, excluding the midvein. We assessed leaf surface area from images captured using a NIKON D5500 DSLR camera (Nikon Co.) mounted on a two‐meter tripod, with plants placed on white paper to prevent background interference. A 15 cm reference ruler was included in each photo for analysis in FIJI‐Image (version 2.9.0). Leaf area was measured three times in ~3‐week intervals during both experiments. At the end of each experiment, we counted the number of leaves per plant and measured the reproductive stalk diameter using a Mitutoyo hand caliper 15 cm above the soil surface.

To evaluate the growth dynamics of each *A. gerardii* population throughout both experiments, we assessed relative growth rate (RGR) for height and leaf area. We used the following equation: RGR = [(height or leaf area at day of experiment *t*)—(height or leaf area at start time = 35 days)]/(time *t*—start time 35 days; Hoffmann and Poorter 2002).

#### 2.1.5 Physiological Traits

To assess plant physiology, we assessed gas exchange, chlorophyll absorbance, and water potential. We measured gas exchange three times during both experiments using a LI‐6400 XT IRGA system (LI‐COR Bioscience, Lincoln, NE, USA). We measured photosynthetic rate, stomatal conductance, water‐use efficiency (WUE; photosynthetic rate/transpiration rate), transpiration rate, and internal CO_2_ concentration. We took measurements from 10:00–14:00 on a young, fully expanded leaf at 400 ppm CO_2_, ambient humidity and temperature, and 1500 μmol photons m^−2^ s^−1^ photosynthetically active radiation. Gas exchange was recorded once photosynthetic rates stabilized and the coefficient of variation remained < 1 for more than 1 min. Each week during both experiments, we assessed leaf chlorophyll absorbance with a SPAD‐502 chlorophyll meter (Konica Minolta; Osaka, Japan), obtaining the mean of at least three readings per plant on fully expanded leaves. We measured midday leaf water potential three times in both experiments using a model 615 pressure chamber (PMS Instrument Co.; Albany, OR, USA). For both experiments, gas exchange and midday leaf water potential measurements were conducted at least 3 h after plants were watered, and this timing was consistent across all populations and treatments.

#### 2.1.6 Phenological Traits

To track phenological changes in both experiments, we monitored plants daily for date of bolting (defined by the presence of a flowering stalk) and flowering (defined by the presence of anthers; Moore et al. 1991) until all plants that did not remain vegetative had flowered.

#### 2.1.7 Statistical Analyses of Functional Traits

##### 2.1.7.1 Trait‐Climate Relationships

For traits measured repeatedly over time, analyses in the main text focus on an average of all time points for the main experiment. For each trait, we fitted separate mixed models for each climate predictor (including linear and quadratic terms) as well as an intercept‐only model. Climate predictors included **l**ongitude, latitude, mean annual precipitation, growing season precipitation, MAT, warmest month temperature, growing season temperature, and coldest month temperature (Table S1). Initially, we assessed maternal effects by including seed mass from each population as a covariate in our models. However, since seed mass was never found to be a significant predictor, it was removed from subsequent analyses. In all models, we used population as a random effect. We also tested for potential block effects (block = plants on the same greenhouse bench); as no effects were significant, blocks were excluded from the final models. We compared models using AIC in JMP pro 17 (SAS Institute Inc., Cary, NC, 1989–2023).

#### 2.1.8 Principal Components Analyses of Trait and Climate Variation

To assess patterns of trait and climate variation across all *A. gerardii* populations in the main experiment, we conducted separate principal components analyses (PCA) on trait data and climate variables. All variables were log‐transformed for standardization prior to analysis. Both PCAs were conducted in JMP Pro 17 (SAS Institute Inc.) using correlation matrices to account for differences in measurement scales.

### 2.2 Genetic Differentiation of *A. gerardii* Populations

To test whether genetic differentiation underlies observed ITV (Obj. 2), we sequenced the source populations using genotyping by sequencing (GBS; Poland and Rife 2012) from in situ individuals sampled across *A. gerardii* populations. Specifically, in 2023, we collected five leaf samples from the midsection of leaves of six plants from the source populations of the main experiment. We flash froze the 150 samples (six plants x 25 populations) on dry ice in the field, freeze dried them for at least 72 h, finely ground the tissue, and stored it at −80**°**C until DNA isolation.

#### 2.2.1 DNA Extraction and Isolation

We isolated DNA using a modified CTAB protocol (Doyle and Doyle 1987). We used 550 μL per sample of extraction buffer added to ground samples containing 100 mM Tris–HCl, pH 8.0, 100 mM EDTA, pH 8.0, 1.4 M NaCl, 2% CTAB, 12 mM TCEP (a non‐toxic substitute for beta‐mercaptanethanol), 16 mM Sodium Diethyldithiocarbamate Trihydrate, 4% PVPP. This was followed by 50 μL per sample of reducing buffer containing 100 mM Tris–HCl, pH 8.0, with 120 mM TCEP and 160 mM DIECA added separately. Quality was quantified for DNA concentration with PicoGreen (ThermoFisher.com) and normalized for library preparation. All samples were prepared for genotyping‐by‐sequencing using a two restriction enzyme pair of PstI and MspI. For each plate, we used 200 base paired‐end sequencing in one Illumina NovaSeq+25b lane.

### 2.3 Genetic PCA and Genetic Differentiation–Trait Association

#### 2.3.1 Genetic PCA

**To** explore individual‐level genetic relationships and background, we conducted a genetic PCA using **SNPRelate** (Zheng et al. 2012) on SNP data derived from the GBS dataset. We applied the PCA to the genotype data to summarize genetic variation among individuals and extracted the first two principal components for visualization and subsequent statistical analyses.

#### 2.3.2 Genetic Differentiation‐Trait Association

To assess the extent to which genetic differentiation explains trait divergence (Obj. 2) in the greenhouse experiment, we fitted a linear mixed‐effects model with Trait PC1 (from the greenhouse trait PCA; Obj. 1) as the response variable and Genetic PC1 (from the genetic PCA) as a fixed effect, with population included as a random intercept. Statistical significance was assessed as *p* < 0.05 on the genetic PCA axis in explaining trait variation. This approach enabled us to relate genetics to trait variation.

### 2.4 Traits in Greenhouse Compared to Field Setting

To compare trait variation between controlled greenhouse and natural field environments (Obj. 3), we used trait data from the same populations of *A. gerardii* that were measured both in the field and here in our greenhouse main experiment. The same set of phenotypic traits was compared in both environmental settings to ensure comparability. To summarize multivariate trait variation across populations, we conducted separate principal component analyses (PCAs) for the greenhouse and field datasets. For the greenhouse experiment, we performed a PCA on the greenhouse trait dataset. For the field comparison, we used PC1 scores from the field PCA reported in Sytsma et al. (2026; data available at Dryad 10.5061/dryad.905qfttzm). We then compared PC1 scores between the greenhouse and field analyses to assess the consistency of population‐level trait differences across these two settings. We log‐transformed variables that did not meet assumptions of normality.

Because PC axis 1 captured a relatively large portion of the variation (68.3% and 56.7% in the field and greenhouse, respectively), we focused on it to compare trait PCA scores between the two settings. To do this, we performed a linear regression between the first principal component axis (PC1) from the greenhouse data and PC1 from the field data. This allowed us to evaluate the extent to which trait variation observed in the greenhouse predicts trait patterns expressed under natural field conditions.

### 2.5 Experimental Drought Effects on Traits

To assess response to experimental drought (Obj 4), we conducted a second experiment with a subset of eight *A. gerardii* populations sourced across a precipitation gradient (472–1356 mm year^−1^ MAP; Figure 1B). The control plants in the drought experiment (Obj. 4) were a separate set of individuals from those used in the main ITV experiment (Obj. 1). The drought experiment had eight replicates (eight populations × two treatments × eight replicate plants = 128 plants total). Initially, plants were watered daily to allow for establishment and early growth. Starting on day 63 of the experiment, watering was reduced to once every 2 days to maintain ~15% soil moisture for the droughted plants. For control plants, watering continued every day to maintain ~30% soil moisture (Figure S2). In the drought experiment, we randomly assigned five permanent probes to five control and five droughted plants and recorded moisture daily (Figure S2). We also took readings of soil moisture from all plants in the experiment (*n* = 128) weekly.

We measured the same morphological, physiological, and phenological traits as in Obj. 1. In addition, to assess biomass allocations in the drought experiment, we harvested aboveground and belowground biomass, stored it in paper bags, and later dried it at 60°C for at least 72 h. Aboveground biomass was subsequently divided into vegetative (leaves), reproductive stalk, or seed allocations and weighed. Belowground biomass was separated into rhizome and root tissue, weighed separately. The aboveground:belowground biomass ratio for each plant was calculated as total aboveground biomass divided by total belowground biomass. To assess biomass allocations in the drought experiment, we harvested aboveground and belowground biomass, stored it in paper bags, and later dried it at 60°C for at least 72 h.

To analyze the effects of drought treatment, population origin, and their interaction on all traits, linear models were implemented in JMP Pro 17. Specifically, we used a factorial linear model with treatment and population origin as fixed factors and their interaction using a two‐way ANOVA. For traits measured repeatedly over time, analyses in the main text focus on a single biologically comparable time point for the drought experiment. Traits were analyzed 38 days after the onset of the drought treatment, when soil moisture in the drought treatment had declined to approximately 15% and treatment effects were clearly expressed.

For each trait, we tested the effects of treatment, population, and their interaction using factorial models. Variance components were estimated by dividing the mean square of each factor by the total variance.

#### 2.5.1 Principal Components Analysis of Trait Responses to Drought

To evaluate how functional trait variation among *A. gerardii* populations responds to drought across a precipitation gradient, we conducted separate PCAs for control and drought treatments. Prior to analysis, all traits were log‐transformed and standardized. Biplots were generated to visualize trait loadings and population‐level variation along the first two principal components. Conducting separate analyses for each treatment allowed us to assess how drought alters multivariate trait coordination and how these changes align with precipitation gradients among population origins.

## 3 Results

### 3.1 Variation in *A. gerardii* Across Broad Climate Gradients

In the main experiment using 25 *A. gerardii* populations (Obj. 1), trait variation was strongly associated with climate‐of‐origin (Table S2). Specifically, 13 of 17 traits had the highest significant association with MAP (*p* < 0.0001) and 4 of 17 traits with growing season temperature (*p* < 0.0001; Table S2).

#### 3.1.1 Morphological Traits

**FIGURE 2:**
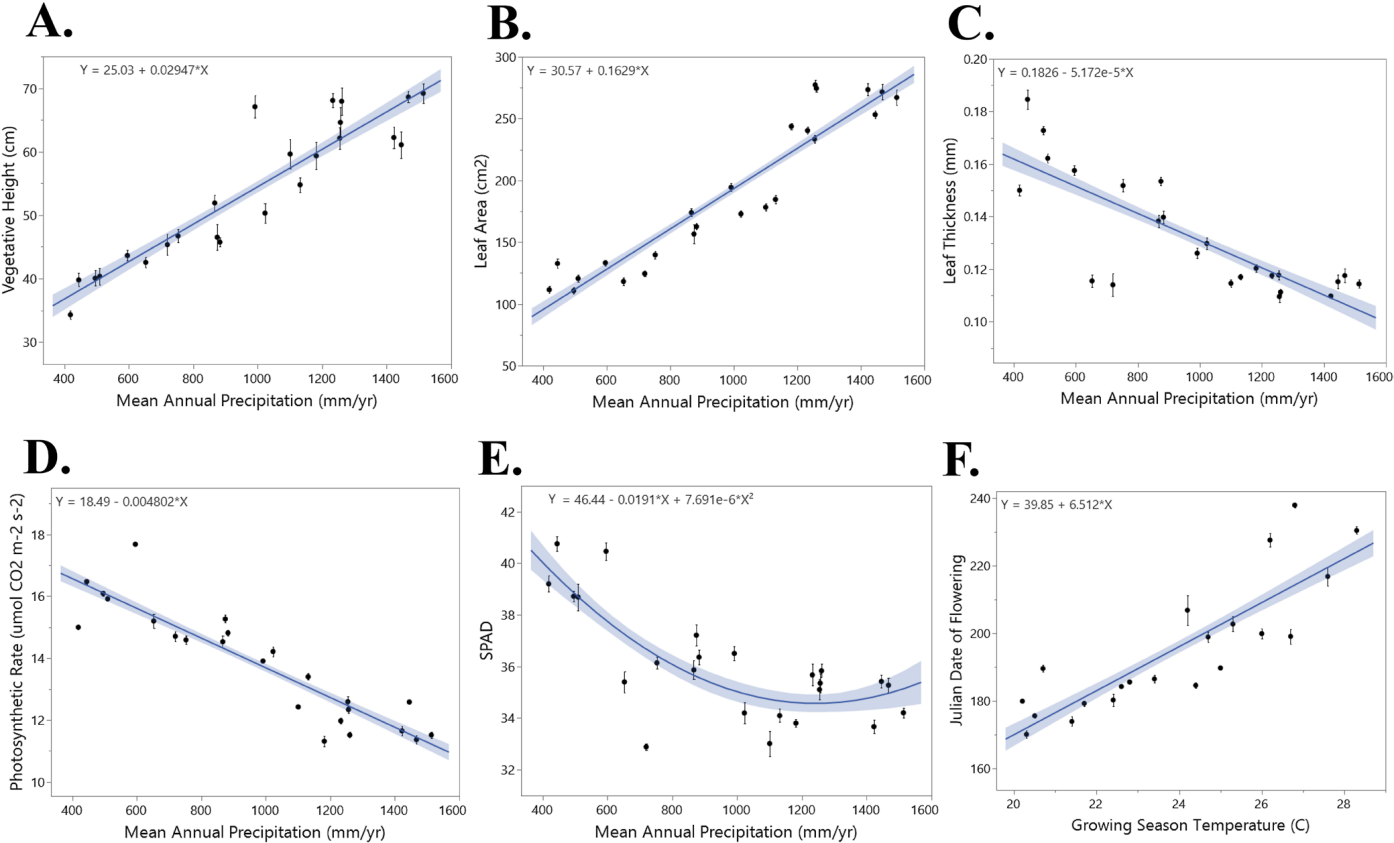
Regressions from the main experiment at mid‐experiment. (A) Vegetative height, (B) leaf area, (C) leaf thickness, (D) photosynthetic rate, and (E) chlorophyll absorbance, and (F) date of flowering. Each trait is regressed over the best predictor by model selection (Table S2). The solid line is the regression, shaded area indicates the confidence interval, points indicate site mean, and error bars indicate site standard error.

Vegetative height increased linearly with MAP (*R* ^2^ = 0.85; *p* < 0.001; Figure 2A), nearly doubling, ranging from ~40 cm in dry to ~75 cm in wet‐origin populations. Also doubling or tripling with MAP were leaf area (~110–225 cm^2^; *R* ^2^ = 0.79; *p* < 0.001), leaf width (range 0.65–1.1 cm; *R* ^2^ = 0.49; *p* < 0.001), leaf number (range 9–25 leaves; *R* ^2^ = 0.71; *p* < 0.001), and stalk diameter (range 2.2–6.9 mm; *R* ^2^ = 0.62; *p* < 0.001; Figure 2B, Figure S3A–C). Leaf thickness increased two‐fold in populations from drier sites (ranging from ~0.19 in dry to ~0.11 mm in wet sites) (*R* ^2^ = 0.62; *p* < 0.001; Figure 2C). Vegetative height growth rates were highest in populations from warmer sites (doubling from cool to warm: 0.24–0.44 cm day^−1^; *R* ^2^ = 0.44; *p* < 0.001). Leaf area growth rate was ~5× greater in populations from cool to warm climates (0.4–2.8 cm^2^/day; *R* ^2^ = 0.53; *p* < 0.001; Figure S3K).

#### 3.1.2 Physiological Traits

Similar to morphological trait responses, all seven physiological traits were most strongly predicted by MAP (Table S2B). The following significantly decreased linearly with precipitation (Figure 2D–E, Figure S3D–G): Photosynthetic rate (range 11.2–18.1 μmol CO₂ m^−2^ s^−1^; *R* ^2^ = 0.48; *p* < 0.001), chlorophyll absorbance (range 31.8–39.7 SPAD units; *R* ^2^ = 0.56; *p* < 0.001), water‐use efficiency (range 3.4–5.3 μmol CO_2_ mmol^−1^ H_2_O, *R* ^2^ = 0.52; *p* < 0.001), water potential (range −0.40 to −0.75 MPa; *R* ^2^ = 0.66; *p* < 0.001), transpiration rate (range 3.5–2.2 mmol H_2_O m^−2^ s^−1^; *R* ^2^ = 0.51; *p* < 0.001; Figure S3E), and stomatal conductance (range 0.139–0.205 mol H_2_O m^−2^ s^−1^; *R* ^2^ = 0.49; *p* < 0.001).

#### 3.1.3 Phenological Traits

In contrast to other traits described above, phenological traits in *A. gerardii* were primarily driven by mean source site growing season temperature (Table S2C). Populations sourced from cooler origins bolted earlier by up to 32 days (*R* ^2^ = 0.68; *p* < 0.001) and flowered earlier by up to 72 days (*R* ^2^ = 0.70; *p* < 0.001; Figure 2F, Figure S3I).

#### 3.1.4 Ordination Analysis

**FIGURE 3:**
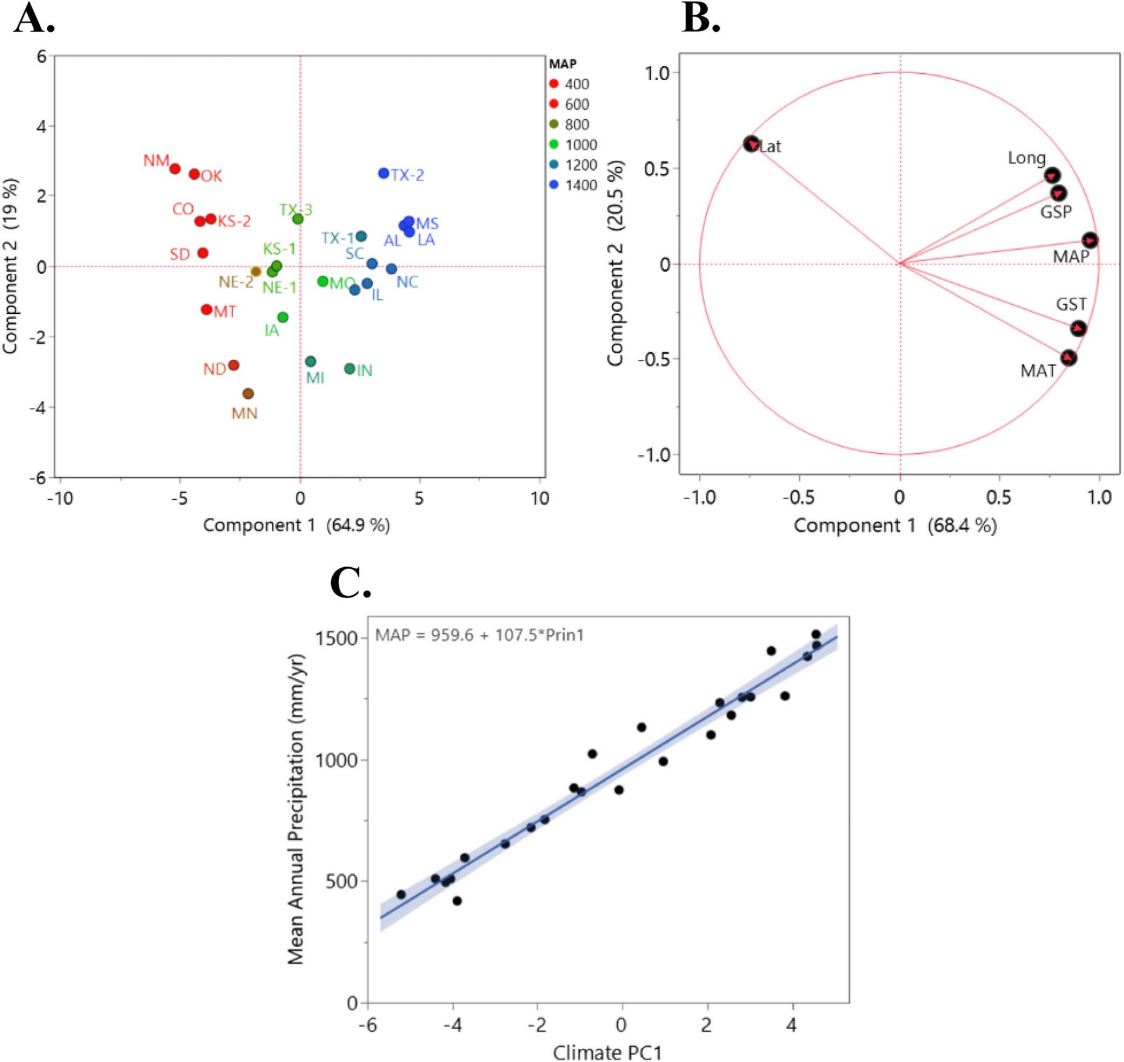
Principal components analyses (PCA) of trait and climate variation across 25 populations. (A) PCA based on climate variables at source locations, illustrating climatic variation across populations. (B) PCA based on morphological, physiological, and phenological trait data from the main greenhouse experiment, showing differentiation among populations along major trait axes. (C) Linear regression showing the relationship between MAP and population scores along trait PCA axis 1. GSP = growing season precipitation, GST = growing season temperature, Lat = latitude, Long = longitude, MAP, mean annual precipitation, MAT, mean annual temperature.

Principal components analysis (PCA) revealed strong climatic structuring of trait variation among *A. gerardii* populations (Figure 3A,B). The first two principal components (PC1 and PC2) explained 64.9% and 19.0% of the total variance, respectively (Figure 3A). Regression analysis confirmed that PC1 scores were tightly aligned with MAP (*R* ^2^ = 0.96, *p* < 0.001; Figure 3C), underscoring precipitation's central role in shaping functional trait divergence across populations.

### 3.2 Genetic Differentiation Explains Variation in Functional Traits

#### 3.2.1 Genetic PCA

**FIGURE 4:**
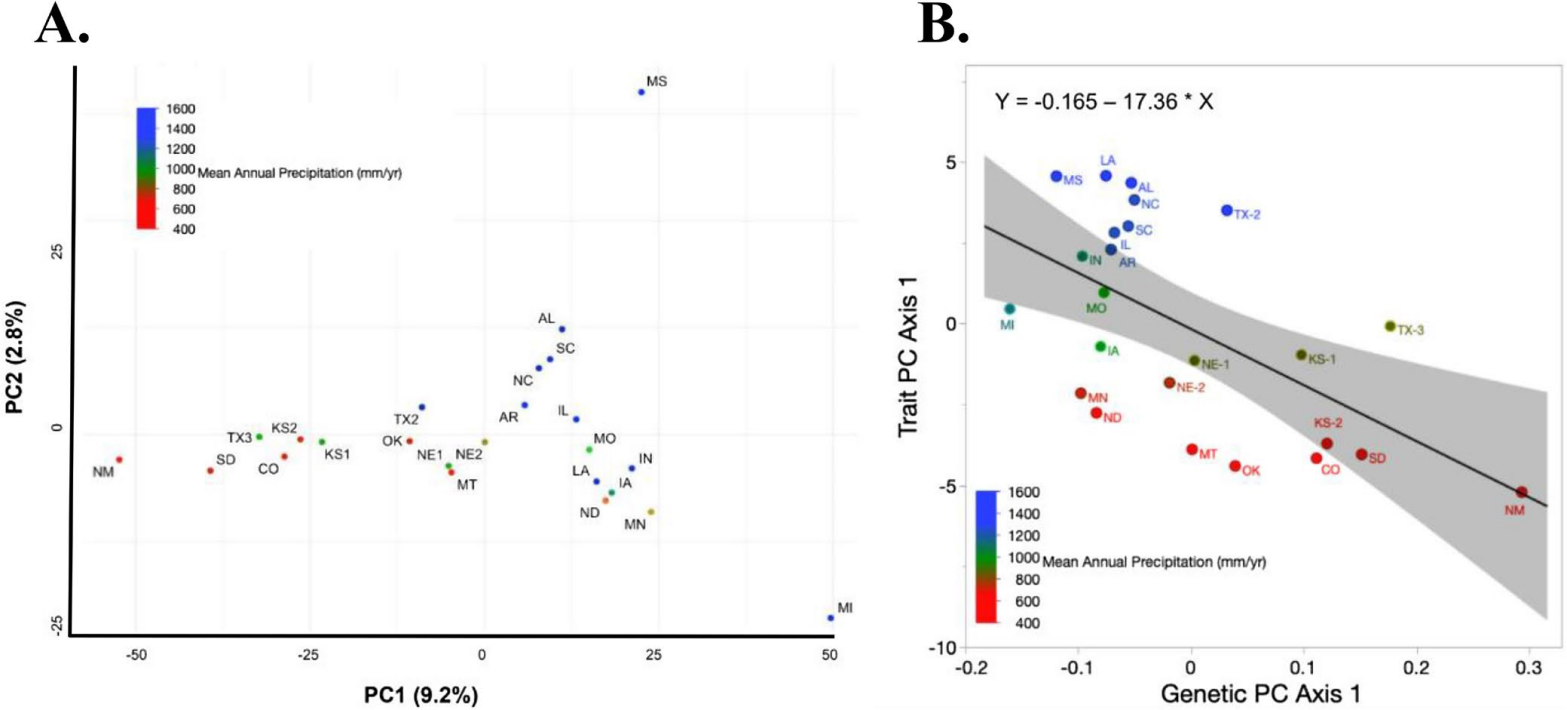
Genetic variation across *A. gerardi* populations. (A) Genetic principal component analysis (PCA) of *A. gerardii* populations across North America, based on SNP profiles. Points indicate averages of populations. (B) Regression analysis of trait PC1 scores across genetic PC1 scores, showing the relationship between genetic differentiation and functional trait variation across populations.

We performed a genetic principal components analysis (PCA) to examine genetic structure across 25 *A. gerardii* populations (Obj. 2; Figure 4A). The first two axes explained 14.3% of the total genetic variance, indicating that a modest proportion of overall genetic variation was captured by these axes. Populations exhibited a tendency to structure along the MAP gradient (Figure 4A), consistent with a partial association between genetic variation and precipitation (linear regression: *R* ^2^ = 0.35).

We also examined the relationship between genetic PC1 scores and trait PC1 scores across populations (Figure 4B). A significant association was found between genetic and trait PC1 scores (*R* ^2^ = 0.35, *p* = 0.002; Figure 4B), indicating genetic differentiation aligns with trait variation across populations. These results suggest that genetic differentiation correlates with ITV across populations, highlighting how genetic differentiation is linked to trait divergence.

### 3.3 Traits Consistent Across Greenhouse and Field Settings

#### 3.3.1 Multivariate Trait Patterns Similar in Field and Greenhouse Settings

**FIGURE 5:**
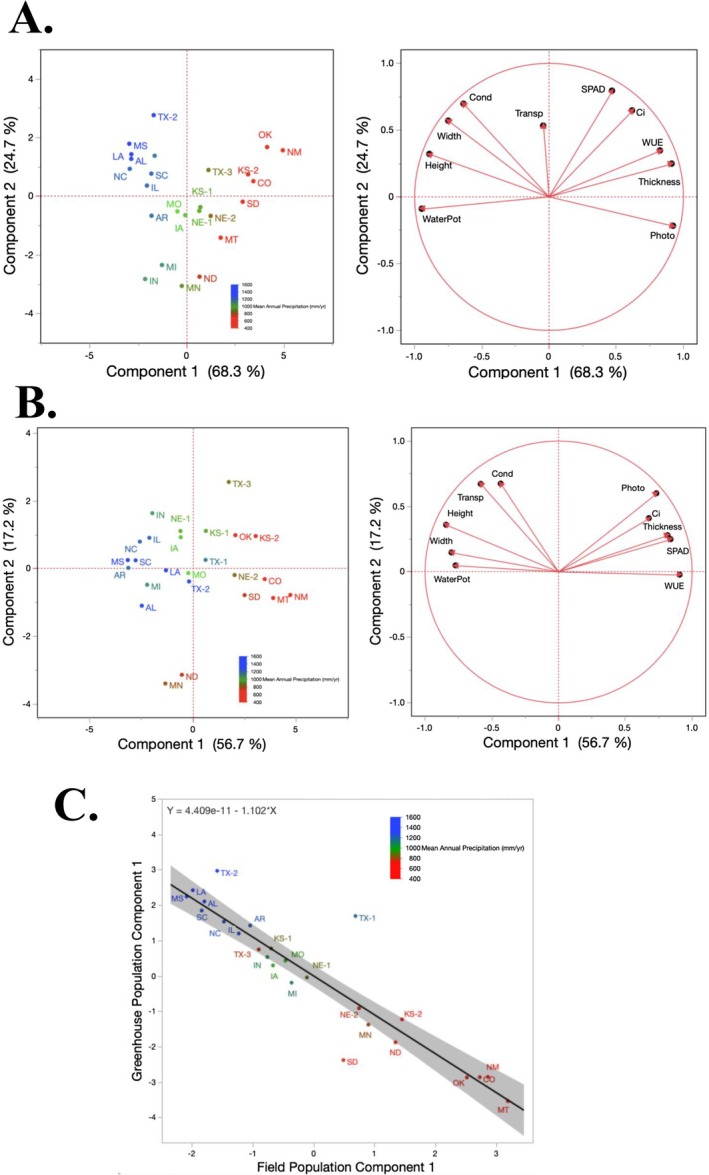
A principal component analysis (PCA) to visualize multivariate trait variation, with the same traits related to morphology and physiology across populations. (A) The main experiment in the greenhouse. (B) The same populations in the field. (C) Linear regression of PCA axis 1 scores from the greenhouse against PCA axis 1 scores from the field, assessing the consistency of population trait patterns across environments. Ci = internal carbon dioxide concentration, Cond = stomatal conductance, Height = vegetative height, Photo = photosynthetic rate, SPAD = chlorophyll absorbance, Thickness = leaf thickness, Transp = transpiration rate, WaterPot = water potential, Width = leaf width, WUE = water use efficiency.

Our PCA revealed distinct patterns of multivariate trait variation among *A. gerardii* populations in both the greenhouse and field settings (Obj. 3). Using only the same traits observed in both studies, our greenhouse trait PCA showed PC1 and PC2 accounted for 68.3% and 24.7% of the variance, respectively (Figure 5A). In the field dataset, PC1 and PC2 accounted for 56.7% and 17.2% of the variance, respectively (Figure 5B).

In both field and greenhouse settings, positive loadings on PC1 were primarily associated with variation in WUE, leaf thickness, internal CO_2_ concentration, and photosynthetic rate (Figure 5A,B). In comparison, variation in water potential, leaf width, and height were related to negative loadings on PC1 in both settings (Figure 5A,B). Together, these results suggest that underlying genetic contributions to trait variation as populations showed consistent separation along PC1 in both field and greenhouse settings.

#### 3.3.2 Comparison of PCA Axes Between Greenhouse and Field Settings

To assess whether trait differences among populations were consistent between field and greenhouse and because PC1 explained over 50% of the variance in both settings, we regressed PC1 scores from the greenhouse and field data sets. We found a significant positive relationship between greenhouse and field population PC1 scores across populations (*R* ^2^ = 0.80, *p* < 0.001; Figure 5C). This indicates that patterns among populations were highly consistent across field and greenhouse and suggests that multivariate trait variation is, in large part, genetically based and consistent across field and greenhouse settings.

### 3.4 Population‐Level Response to Experimental Drought: Wet‐Origin Populations of *A. gerardii* Are More Affected by Drought

In the drought experiment (Obj. 4), most traits exhibited significant responses to experimental drought. We analyzed each trait using a factorial linear model including block, treatment, population, and the treatment × population interaction. These analyses indicated significant main effects of drought for the majority of traits examined, whereas leaf width and leaf thickness showed no significant responses (Tables S3 and S4). Significant treatment × population interactions were detected for most functional traits, indicating that the magnitude of drought responses differed among populations across the precipitation gradient (Table S3).

#### 3.4.1 Morphological Traits

**FIGURE 6:**
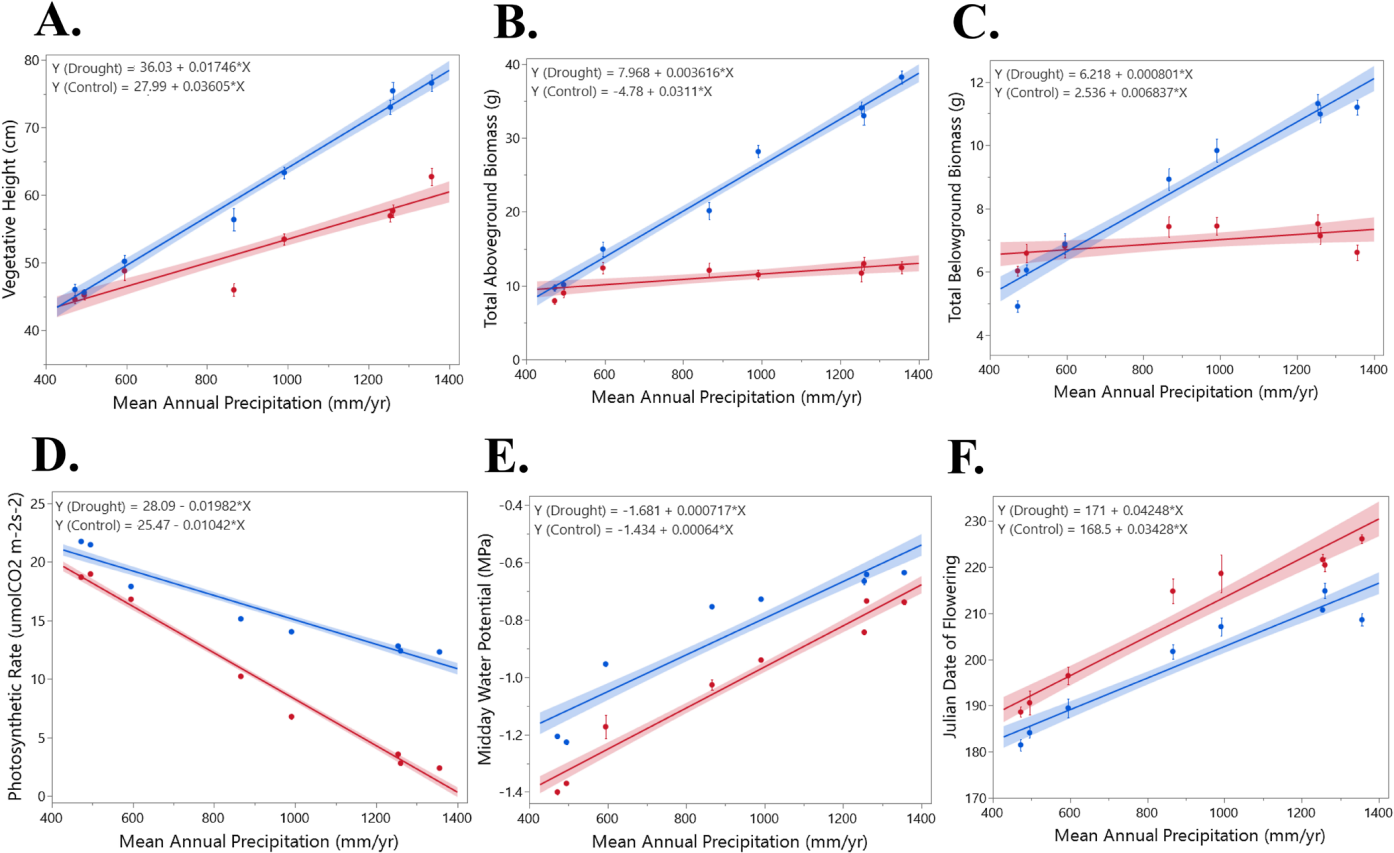
Regressions from the drought experiment, 38 days after the onset of drought. (A) Plant height, (B) total aboveground biomass, (C) total belowground biomass, (D) photosynthetic rate, (E) midday water potential, and (F) date of flowering across a mean annual precipitation gradient. Blue indicates control and red indicates drought. The solid line is the regression, shaded area indicates the confidence interval, points indicate site mean, and error bars indicate site standard error.

Under experimental drought (Figure 6), we observed significant reductions in several morphological traits across *A. gerardii* populations. Specifically, ANOVA results showed strong main effects of drought treatment for vegetative height ($F_{1,105}=145.2$, p<0.001), leaf area ($F_{1,105}=32.8$, p<0.001), total aboveground biomass ($F_{1,105}=128.6$, p<0.001), and total belowground biomass ($F_{1,105}=97.4$, p<0.001; Figure 6A–C; Tables S3 and S4). Significant treatment × population interactions were also detected for these traits, including vegetative height ($F_{7,105}=6.84$, p<0.001), leaf area ($F_{7,105}=4.12$, p=0.001), total aboveground biomass ($F_{7,105}=5.97$, p<0.001), and total belowground biomass ($F_{7,105}=4.88$, p<0.001), indicating population‐specific responses to drought.

Additional morphological traits showed similar drought‐associated declines, including number of leaves ($F_{1,105}=81.3$, p<0.001), stalk diameter ($F_{1,105}=72.4$, p<0.001), vegetative biomass ($F_{1,105}=18.9$, p<0.001), reproductive biomass ($F_{1,105}=93.5$, p<0.001), seed biomass ($F_{1,105}=21.4$, p<0.001), and root biomass ($F_{1,105}=89.7$, p<0.001) (Figure S4A–J; Tables S3 and S4). Significant treatment × population interactions were also detected for most of these traits, including number of leaves ($F_{7,105}=5.16$, p<0.001), stalk diameter ($F_{7,105}=4.73$, p<0.001), vegetative biomass ($F_{7,105}=3.21$, p=0.004), reproductive biomass ($F_{7,105}=5.48$, p<0.001), seed biomass ($F_{7,105}=3.67$, p=0.002), and root biomass ($F_{7,105}=4.94$, p<0.001). In contrast, leaf width ($F_{1,105}=1.32$, p=0.25; treatment × population: $F_{7,105}=1.08$, p=0.38) and leaf thickness ($F_{1,105}=0.87$, p=0.35; treatment × population: $F_{7,105}=1.14$, p=0.35) did not respond significantly to drought treatment across populations.

Population‐level differences in drought sensitivity were evident. Populations originating from wetter climates exhibited the strongest declines across most morphological traits, whereas populations from drier climates showed comparatively small changes under drought conditions. Under drought treatment, wet‐origin populations showed reductions in height (~28%), leaf area (~48%), aboveground biomass (~27%), belowground biomass (~47%), number of leaves (~42%), stalk diameter (~46%), vegetative biomass (~37%), reproductive biomass (~39%), seed biomass (~72%), and root biomass (~36%) relative to control conditions (Figure 6, Figure S4A–J). In contrast, dry‐origin populations exhibited minimal reductions, typically less than 5% across morphological traits.

Vegetative growth rates, estimated from changes in plant height and leaf area, were also significantly reduced under drought treatment (height growth: $F_{1,105}=54.6$, p<0.001; leaf area growth: $F_{1,105}=61.9$, p<0.001; Table S3). Significant treatment × population interactions indicated that these declines varied among populations (height growth: $F_{7,105}=4.41$, p<0.001; leaf area growth: $F_{7,105}=4.76$, p<0.001). Growth rate declines were most pronounced in populations originating from wetter climates, whereas populations from drier climates exhibited little change under drought (Figure S4R–S).

#### 3.4.2 Physiological Traits

Physiological traits also responded strongly to experimental drought. ANOVA models revealed significant main effects of drought for photosynthetic rate ($F_{1,105}=110.3$, p<0.001), stomatal conductance ($F_{1,105}=96.8$, p<0.001), water‐use efficiency ($F_{1,105}=44.7$, p<0.001), and chlorophyll absorbance ($F_{1,105}=87.1$, p<0.001) (Figure 6D, Figure S4L–P; Tables S3 and S4). Significant treatment × population interactions were also detected for these physiological traits, including photosynthetic rate ($F_{7,105}=6.12$, p<0.001), stomatal conductance ($F_{7,105}=5.55$, p<0.001), water‐use efficiency ($F_{7,105}=3.84$, p=0.001), and chlorophyll absorbance ($F_{7,105}=4.97$, p<0.001), indicating variation in drought sensitivity among populations.

As observed for morphological traits, the strongest physiological declines occurred in populations originating from wetter environments. Wet‐origin populations exhibited large drought‐associated reductions in photosynthetic rate (~80%), stomatal conductance (~68%), water‐use efficiency (~65%), and chlorophyll absorbance (~48%) relative to control conditions (Figure 6, Figure S4L–P). In contrast, dry‐origin populations maintained relatively stable physiological performance under drought, with changes generally less than 10%.

#### 3.4.3 Phenological Traits

Experimental drought also significantly altered reproductive timing. ANOVA models indicated significant effects of drought treatment on bolting time ($F_{1,105}=29.7$, p<0.001) and flowering time ($F_{1,105}=73.5$, p<0.001) (Figure 6F, Figure S4Q; Tables S3 and S4). On average, drought delayed bolting by approximately 5 days and flowering by approximately 9 days across populations. Significant treatment × population interactions indicated that the magnitude of these phenological delays varied among populations, with the strongest delays observed in populations originating from wetter climates (bolting: $F_{7,105}=3.46$, p=0.002; flowering: $F_{7,105}=4.28$, p<0.001).

#### 3.4.4 Ordination Analysis

**FIGURE 7:**
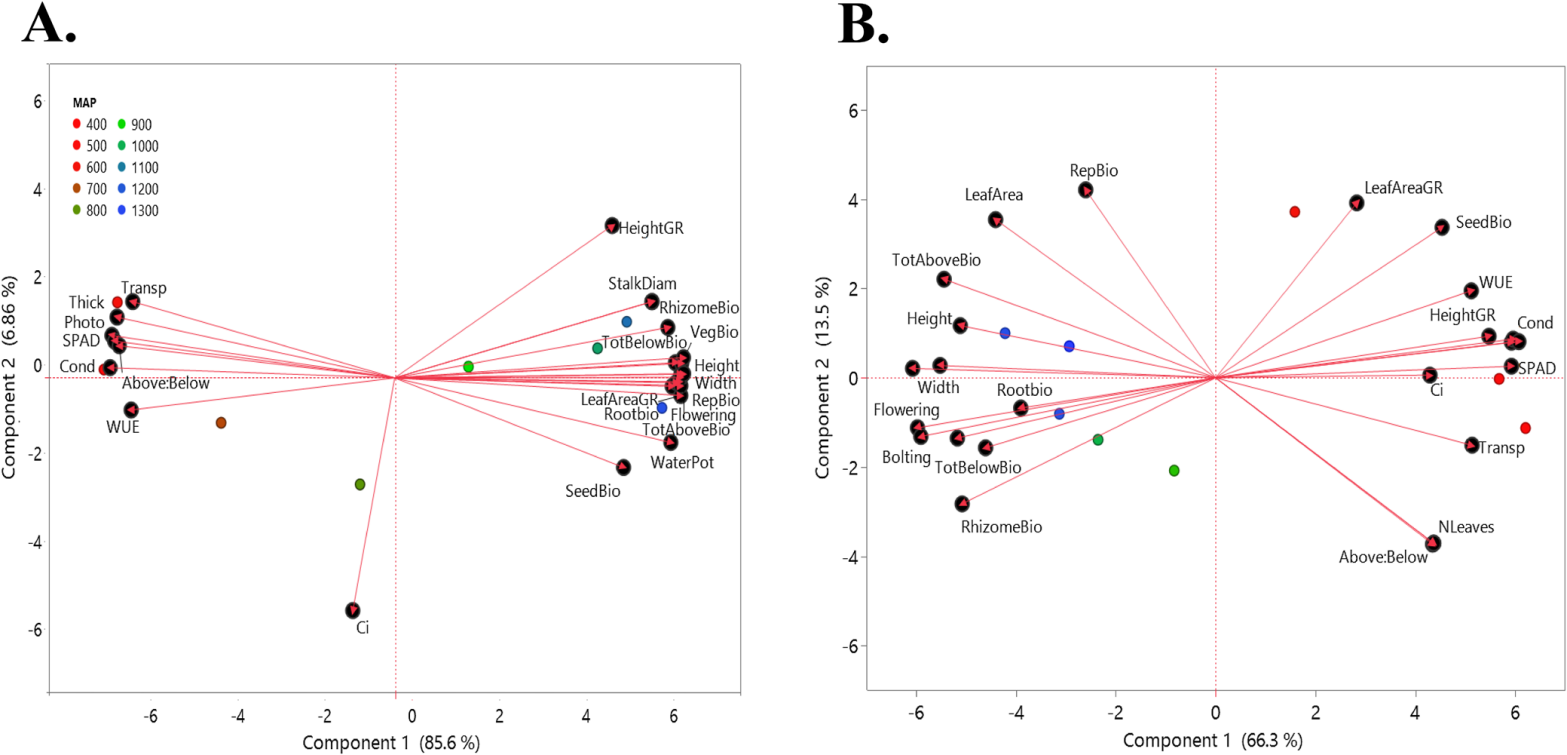
Principal Components Analysis (PCA) of trait variation across a precipitation gradient under control and drought treatments. (A) PCA biplot of trait data from the control treatment, showing population‐level variation in functional traits across the precipitation gradient. (B) PCA biplot of trait data from the drought treatment, illustrating shifts in trait expression under water limitation. Arrows indicate loadings of key traits contributing to variation along PCA axes. Above:Below = aboveground: belowground biomass, Bolting = date of bolting, Ci = internal carbon dioxide concentration, Flowering = date of flowering, Height = vegetative height, HeightGR = height‐based growth rate, LeafAreaGR = leaf area based growth rate, Photo = photosynthetic rate, RepBio = reproductive biomass, RhizomeBio = rhizome biomass, RootBio = root biomass, SeedBio = seed biomass, SPAD = chlorophyll absorbance, StalkDiam = stalk diameter, Thick = leaf thickness, TotBelowBio = total belowground biomass, Transp = transpiration rate, VegBio = vegetative biomass, WaterPot = water potential, Width = leaf width, WUE = water use efficiency.

Our PCA revealed distinct patterns of trait variation across the precipitation gradient under both control and drought treatments (Figure 7). In the control treatment (Figure 7A), *A. gerardii* populations exhibited a spread along PCA axis 1, where positive loadings on PC1 were primarily associated with morphological traits such as increased height, biomass, and leaf area. In contrast, negative loadings were associated with dry‐origin populations and with traits such as WUE, leaf thickness, chlorophyll absorbance, and photosynthetic rate, suggesting differences in growth and allocation patterns among populations (Figure 7A).

Under drought conditions (Figure 7B), populations shifted in PCA space, where positive loadings on PC1 were associated with dry‐origin populations, including grouping traits such as number of leaves, seed biomass, growth rate, and stomatal conductance. Negative loadings on PC1 were associated with wet‐origin populations, including traits such as flowering, bolting, leaf width, and height (Figure 7B).

## 4 Discussion

Our study demonstrates that, among the climatic gradients evaluated, precipitation has imposed the strongest selection pressure across the natural range of *A. gerardii* , emerging as a fundamental driver of phenotypic divergence across broad MAT (4°C–21°C) and precipitation (350–1400 mm year^−1^) gradients. Overall, results highlight the importance of adaptation to historical climate‐of‐origin in shaping functional trait variation (Wadgymar et al. 2022). Here, we explore four key aspects in detail: First, we consider how climate‐of‐origin, in particular precipitation, shapes ITV across populations of this widespread foundation plant species. Second, we reveal how climate‐associated genetic divergence contributes to population differentiation in trait variation, and how this genetic differentiation aligns with patterns of ITV. Third, we discuss how similar patterns of ITV from the same populations across greenhouse and field settings reveal consistent population‐level differentiation, providing evidence that ITV is largely genetically determined. Fourth, we discuss how populations differ in their physiological, morphological, and phenological responses to experimental drought, revealing shifts in trait patterns under water‐limited conditions, with wet‐origin populations showing greater changes in functional traits while dry‐origin populations perform similarly across hydric scenarios. Finally, we explore the implication of this work for conservation and restoration.

### 4.1 Trait Variation Among *A. gerardii* Populations

Our results demonstrate that long‐term climatic gradients, particularly in precipitation, have played a central role in shaping ITV (Siepielski et al. 2017; Feldman et al. 2024) in *A. gerardii* . PCA analyses (Figure 3) revealed consistent differences between populations from wetter and drier environments, indicating long‐term rainfall shapes functional patterns. For example, populations from wet sites exhibited acquisitive traits such as taller stature, broader and more numerous leaves, and greater leaf area (Jardine et al. 2021), whereas populations from dry sites expressed conservative traits—shorter stature, reduced leaf area, and thicker leaves—adapted for water conservation (Moles et al. 2009; Wang et al. 2022). These coordinated trait shifts are consistent with climate‐associated divergence and potential adaptive differentiation, reflecting integrated trait patterns shaped by long‐term climatic conditions. Similar findings have been documented in other C_4_ grasses, confirming the repeated emergence of conservative trait syndromes such as reduced leaf area, conservative growth, and efficient water use under dry conditions (Taylor et al. 2014; Lowry et al. 2014; Aspinwall et al. 2017; Griffin‐Nolan et al. 2025; Heckman et al. 2025).

Physiological traits also were related to precipitation where dry‐origin populations showed higher water‐use efficiency and increased chlorophyll absorbance. Interestingly, dry‐origin populations also exhibited higher gas exchange rates under well‐watered conditions, which may reflect greater baseline photosynthetic capacity (Fernández‐de‐Uña et al. 2023) or a strategy of rapid carbon gain when water is available (Niinemets 2007). These patterns are consistent with broader evidence that physiological traits such as water‐use efficiency, chlorophyll content, and gas exchange are closely linked to precipitation regimes and can influence plant performance under variable water availability (Galmés et al. 2007; Niinemets 2007; Fernández‐de‐Uña et al. 2023). Such physiological adjustments highlight the role of plasticity in facilitating persistence across heterogeneous environments (Brooker et al. 2022).

Phenological traits such as bolting and flowering time were influenced primarily by source site temperature (Figure 2F). Populations of *A. gerardii* from cooler climates flowered over 2 months earlier than those sourced from warmer climates. This early flowering is likely an adaptation to shorter growing seasons going from wet to dry (Johansson et al. 2013; Bhattacharya 2022). Earlier phenological transitions in cooler‐origin populations mirror adaptations to short growing seasons documented in other C_4_ grasses (McMillan 1956, 1961; Lovell et al. 2021; Heckman et al. 2025), but not to the extent we document here with a 72‐day difference in flowering time between the coolest and warmest *A. gerardii* populations. This pronounced difference in flowering time of *A. gerardii* populations could indicate emerging reproductive isolation (Baack et al. 2015).

Collectively, these results provide a coherent historical framework linking climatic gradients to coordinated morphological, physiological, and phenological divergence in *A. gerardii* . Overall, our results highlight how *A. gerardii* populations exhibit coordinated morphological, physiological, and phenological trait variation in response to local climate, illustrating the multifaceted ways environmental gradients drive ITV.

### 4.2 Genetic Differentiation Explains Trait Divergence Among *A. gerardii* Populations

In our study, genetic PCA revealed strong and geographically structured genetic differentiation among *A. gerardii* populations (Figure 4). This genetic structure aligned closely with patterns of trait variation, as shown by a significant correlation in a regression between genetic and trait PC scores, indicating congruence between genetic and phenotypic differentiation. This suggests that genetic differentiation among populations may reflect responses to spatially varying selective pressures (Chung et al. 2023), particularly variation in precipitation. Similar associations between genetic and phenotypic divergence have been documented in a range of species (Jarvis et al. 2025; Buenaño et al. 2025), including *A. gerardii* (Gray et al. 2014; Johnson et al. 2015; Galliart et al. 2019, 2020), supporting climate in shaping genetic differentiation.

The modest alignment of genetic axes with trait variation suggests that populations in different climate regimes may have undergone selection for distinct trait syndromes optimized for local conditions (Le Corre and Kremer 2012; Savolainen et al. 2013; Sork 2018). Similar genotype–phenotype associations have been observed in other species where climatic variables like precipitation and temperature strongly predict both genetic structure and trait differentiation (Galliart et al. 2019, 2020; Blanco‐Sánchez et al. 2024). These patterns highlight the role of climate‐associated variation in maintaining genetic diversity across wide geographic ranges (Lait et al. 2025). Such a pattern reinforces the idea that climate‐related selective pressures can leave detectable signatures in both the genome and phenotype (Savolainen et al. 2013; Lowry et al. 2015; Lovell et al. 2021).

The alignment between genetic and trait PCA scores further indicates that precipitation contributes modestly to genetic differentiation among populations and supports a genetic basis for ITV. Additional micro‐environmental factors, such as local soil conditions, fine‐scale variation in soil moisture and nutrients, and plant–microbe interactions in the rhizosphere, may also contribute to the remaining genetic and phenotypic variation unexplained by our PCA analyses (Kazarina et al. 2023; Govaert et al. 2024). Taken together, these results demonstrate that both climatic gradients and genetic differentiation contribute to shaping phenotypic diversity within *A. gerardii* . Together, these insights emphasize that preserving genetic and phenotypic diversity across climate gradients is essential for sustaining the adaptive potential of species (Bomblies and Peichel 2022) under predicted droughts. By establishing this climatic and genetic context first, our findings clarify how long‐term environmental pressures shape ITV and set the stage for understanding population‐specific responses to contemporary drought stress.

### 4.3 Traits Align in Greenhouse and Field Settings, Confirming Genetic Basis for Trait Variation

By comparing ITV in the greenhouse common garden and the field for the same *A. gerardii* populations, we found consistent population‐level differentiation in trait variation across environments. Multivariate analyses showed similar covariation among key traits in both settings, and population‐level trait patterns were strongly aligned between greenhouse and field PCAs (Figure 5), indicating that genetic variation contributes meaningfully to ITV by maintaining population‐specific trait syndromes across contrasting environments.

The coordinated covariation of traits such as water‐use efficiency, leaf thickness, and photosynthetic rate (Figure 5) reflects integrated physiological trait patterns associated with contrasting climates (Grime 1988; Jardine et al. 2021). Because instantaneous gas exchange rates can be strongly influenced by short‐term environmental conditions, these multivariate trait relationships provide a more integrative measure of plant functional responses across environments. The consistency of these patterns between greenhouse and field conditions suggests underlying genetic differentiation aligned with climatic gradients (Anderson et al. 2011; Wadgymar et al. 2022; Bomblies and Peichel 2022; Juenger 2013; Sork 2018; Schwinning et al. 2022).

Despite the more complex biotic interactions in the field (Collins et al. 2022; de la Torre Cerro and Holloway 2021), trait alignment between greenhouse and field plants remains strong (Figure 5C,D), reinforcing the role of genetic rather than environmental control. This consistency also demonstrates that common garden experiments, at least for this species, can capture genetically based trait variation relevant to natural conditions, highlighting the value of integrating controlled and field studies for understanding plant adaptation (Schwinning et al. 2022). However, because plants were grown from seed, some correspondence between greenhouse and field trait variation may reflect maternal effects. However, despite the more complex biotic interactions present in field environments (Collins et al. 2022; de la Torre Cerro and Holloway 2021), the strong alignment between greenhouse and field trait patterns (Figure 5C,D) suggests that genetic differentiation, potentially alongside early‐life effects, contributes to population‐level trait structure.

This genetic differentiation offers a mechanistic explanation for the well‐described patterns of grassland productivity decline along precipitation gradients across the Great Plains (Weaver 1954; Sala et al. 1988; Epstein et al. 1998). Although dry‐origin populations exhibited higher photosynthetic rates at the leaf level under well‐watered conditions, they nevertheless produced lower overall biomass. This apparent mismatch suggests that instantaneous carbon assimilation does not necessarily translate directly into greater whole‐plant productivity, as differences in plant size, growth rates, and biomass allocation can constrain total carbon gain. Dry‐origin populations may therefore maintain relatively high leaf‐level carbon capture while exhibiting more conservative growth patterns that limit aboveground biomass accumulation. In this way, declining productivity of *A. gerardii* toward more arid regions likely reflects climate‐associated genetic differentiation shaping growth potential, rather than short‐term water limitation alone.

### 4.4 Precipitation Is the Main Selective Pressure in Population‐Specific Drought Response

In our drought experiment, *A. gerardii* populations differed markedly in response: those from wetter climates showed strong trait shifts under water stress, whereas populations from drier regions were minimally affected and maintained more conservative, drought‐resilient traits (Figures 6 and 7). These contrasting responses suggest not only fixed trait differences but also variation in phenotypic plasticity (Stotz et al. 2021). Specifically, wet‐origin populations expressed greater plasticity under drought, but this came at a cost of reduced performance, likely reflecting genetic constraints on tolerance (Juenger 2013; Benning et al. 2023). In contrast, dry‐origin populations exhibited limited plasticity, likely since they are already pre‐adapted to low rainfall, maintaining function under stress with little adjustment. Thus, drought responses are not uniform but depend on climate‐of‐origin, with dry‐origin populations showing fixed adaptive traits and wet‐origin populations relying more on plasticity (De Kroon et al. 2005; Matesanz and Ramírez‐Valiente 2019; Hoffman and Smith 2021).

Ordination analyses further indicated that drought influenced not only trait magnitudes but also the overall structure of trait relationships among populations. Under drought treatment, dry‐origin populations generally maintained growth‐related traits such as stomatal conductance and leaf number, indicating relatively stable functional performance. In contrast, wet‐origin populations exhibited stronger shifts in trait expression, including delayed flowering and reduced plant size (Figure 6). These patterns suggest that drought primarily altered trait coordination and expression in populations from wetter climates, whereas dry‐origin populations maintained more stable trait profiles under water limitation. This pattern suggests that populations from drier sites may exhibit trait combinations consistent with greater drought tolerance (Gupta et al. 2020), while populations from wetter sites show acclimatory trait adjustments under reduced rainfall (Brunet et al. 2025).

This drought‐induced reorganization of trait assemblages reflects a common shift in functional traits across populations, characterized by reduced investment in aboveground growth and reproduction and increased allocation to belowground and efficiency‐related traits. The trait assemblages also indicated coordinated declines in aboveground and reproductive biomass, consistent with reduced investment in reproductive stalks and seed production and relatively greater allocation to rhizomes and roots under drought (Table S3; Figure S4). Such shifts in biomass allocation may enhance resource conservation and water acquisition, allowing plants to maintain survival and persistence during periods of water limitation. Although this reorganization occurred across all populations, shifts were more pronounced in wet‐origin populations, suggesting stronger plastic responses rather than greater drought sensitivity per se (Gazol et al. 2023).

Experimental drought also delayed bolting and flowering across all populations. Because sexual reproduction maintains genetic variability within populations (Heckman et al. 2025), prolonged delays in flowering under drought conditions could potentially reduce opportunities for reproduction and, over time, influence levels of ITV. Similar patterns of delayed reproductive phenology have been observed in field observations of natural and experimental prolonged drought in grasslands (Schuchardt et al. 2021). Taken together, these results confirm precipitation as a key selective agent underlying ITV in *A. gerardii* and demonstrate that adaptation to rainfall shapes coordinated trait reorganization under drought stress.

### 4.5 Implications for Conservation and Restoration Under Predicted Droughts

The results from this study have implications for future climate scenarios, where more frequent and intense drought events (IPCC 2023; King et al. 2024) could alter the distribution of species (Aldea et al. 2024) and populations (Krishnadas et al. 2021), particularly those from wetter regions (Craine et al. 2013). We found that climate‐of‐origin not only shapes trait variation but ultimately also determines underlying genetic structure and adaptive functional trait organization. Understanding these trait‐genetic relationships is vital for predicting plant responses to future drought stress. Populations with greater drought tolerance may have a better chance of surviving in more water‐limited environments, potentially driving shifts in plant communities across climatic gradients (Krishnadas et al. 2021).

In addition, our results also help resolve a long‐standing question regarding why *A. gerardii* productivity declines along the east–west precipitation gradient of the US Great Plains (Weaver 1954; Sala et al. 1988; Epstein et al. 1998). By comparing trait variation under field and common garden conditions and linking these patterns to genetic differentiation, we show that productivity declines in drier regions are not solely the result of short‐term physiological plasticity approaching environmental limits. Instead, populations from drier climates consistently expressed conservative trait syndromes under well‐watered conditions, indicating a genetic component (Sork 2018) to reduced stature and growth. At the same time, plastic responses to drought were evident across all populations, suggesting that both genetic differentiation and phenotypic plasticity contribute to productivity patterns across the rainfall gradient.

Importantly, the observed patterns of ITV were also correlated with population‐level genetic differentiation. This highlights the need to incorporate ITV and genetic background into conservation and restoration planning. For long‐lived species like *A. gerardii* , strategies such as introducing genotypes from populations adapted to projected future climates (Vitt et al. 2022) could enhance resilience and ensure restored populations are better equipped to cope with changing precipitation and temperature regimes (Hord et al. 2025). Doing so will improve the selection of resilient plant populations, helping to sustain grassland ecosystems under future droughts. Since *A. gerardii* is a foundation grass species in tallgrass prairies (Knapp et al. 1998), its response to experimental drought provides key insights into how the prairie as a whole may respond to drought (Gitlin et al. 2006; Zhang et al. 2025). Therefore, understanding the mechanisms of drought tolerance in *A. gerardii* can inform broader predictions of how grasslands may respond to drought.

## 5 Conclusion

This study demonstrates the strong influence of climate of origin on functional trait variation in *A. gerardii* across broad temperature and precipitation gradients. Our results show that precipitation‐related climatic conditions play a major role in shaping ITV and responses to experimental drought. These patterns are associated with genetically based differences among populations, indicating that long‐term environmental variation has contributed to functional divergence across the species' geographic range. Our findings also provide a potential explanation for the well‐documented east–west decline in grass productivity across the Great Plains. Populations originating from drier regions remained smaller and less productive even under well‐watered greenhouse conditions, suggesting that population‐level differences in growth potential reflect genetically based differentiation rather than short‐term phenotypic responses. Understanding how population origin influences trait variation and drought responses is important for predicting plant performance under increasing drought frequency. These results highlight the importance of considering population‐level variation when selecting plant material for restoration and management under future climatic conditions.

## Author Contributions

**Jack Sytsma:** conceptualization (equal), data curation (equal), formal analysis (lead), investigation (lead), methodology (equal), supervision (equal), writing – original draft (lead). **Helen Winters:** investigation (equal), writing – review and editing (equal). **Matthew Galliart:** investigation (equal), writing – review and editing (equal). **Ryann Patterson:** investigation (equal), writing – review and editing (equal). **Brian Maricle:** validation (equal), writing – review and editing (equal). **Loretta Johnson:** conceptualization (equal), funding acquisition (equal), methodology (equal), project administration (equal), resources (equal), writing – review and editing (equal).

## Funding

This work was supported by the U.S. Department of Agriculture, Agricultural Research Service (award number: 2020‐03665).

## Conflicts of Interest

The authors declare no conflicts of interest.

## Supporting information


**Figure S1.** Soil moisture over time in the main experiment. Data are presented as daily averages, and the line indicates the trend line. The plot illustrates the variation in soil moisture over the course of the experiment and remaining mostly near 30% soil moisture throughout the main experiment.
**Figure S2.** A. Soil moisture over time in the drought experiment. Data are presented as daily averages, and the line indicates the trend line highlighting the differences in moisture availability between control (mean = 30.5%) and drought (mean = 15.2%) treatments after start of drought until the end of the experiment.
**Figure S3.** Regressions from the main experiment mid‐experiment. (A) Leaf width, (B) number of leaves, (C) stem diameter, (D) stomatal conductance, (E) transpiration rate, (F) water use efficiency, (G) internal carbon dioxide concentration, (H) water potential, (I) date of bolting, (J) relative height growth rate and (K) relative leaf area growth rate. The solid line is the regression, shaded area indicates the confidence interval, points indicate site mean, and error bars indicate site standard error. The equation of the line of best fit is included.
**Figure S4.** Regressions from the drought experiment. (A) Leaf area, (B) number of leaves, (C) stem diameter, (D) leaf thickness, (E) leaf width, (F) vegetative biomass, (G) reproductive biomass, (H) seed biomass, (I) rhizome biomass, (J) root biomass, (K) root to shoot ratio, (L) stomatal conductance, (M) transpiration rate, (N) water‐use efficiency, O. carbon dioxide concentration, (P) chlorophyll absorbance, (Q) date of bolting, (R) height‐based growth rate, and (S) leaf area‐based growth rate across a precipitation gradient. Blue indicates control and red indicates drought. The solid line is the regression, shaded area indicates the confidence interval, points indicate site mean, and error bars indicate site standard error. The equation of the line of best fit for each treatment (control or drought) is included.
**Table S1A.** Site information, location (longitude and latitude), and home climate variables showing historic annual temperature and precipitation and other relevant site details.
**Table S1B.** Site information with home site soil moisture, temperature, pH, and texture.
**Table S2A.** The results of regression analysis performed to analyze the effects of home on morphological trait variation in the main experiment. The most explanatory climate predictor is in bold for each trait.
**Table S2B.** The results of regression analysis performed to analyze the effects of home on physiological trait variation in the main experiment. The most explanatory climate predictor is in bold for each trait.
**Table S2C.** The results of regression analysis performed to analyze the effects of home on phenological and growth‐related trait variation in the main experiment. The most explanatory climate predictor is in bold for each trait.
**Table S2D.** The results of regression analysis performed to analyze the effects of home on physiological, morphological, and phenological traits reporting the variance explained by *R* ^2^m and *R* ^2^c.
**Table S3.** The results of general linear models (GLMs) performed to analyze the effects of drought treatment, population origin, and their interaction on trait variation. Significant population × treatment interaction in various time points predrought or after start of drought are in bold for each response variable.
**Table S4A.** The results of general linear models (GLMs) of morphological traits performed to analyze the effects of drought treatment, population origin, and their interaction on trait variation over time (e.g., Drought T2 = the effect of drought treatment on time period = 2).
**Table S4B.** The results of general linear models (GLMs) of physiological traits performed to analyze the effects of drought treatment, population origin, and their interaction on trait variation over time (e.g., Drought T2 = the effect of drought treatment on time period = 2).
**Table S4C.** The results of general linear models (GLMs) of phenological and growth‐related traits performed to analyze the effects of drought treatment, population origin, and their interaction on trait variation over time (e.g., Drought T2 = the effect of drought treatment on time period = 2).

## Data Availability

All data supporting the findings of this study are available in a publicly accessible Dryad repository: https://doi.org/10.5061/dryad.1vhhmgr71.
