# Supplementary material for: Drivers of Intraspecific Trait Variation and Drought Response of a Dominant US Great Plains Grass: Disentangling the Role of Climate and Genetic Background
Source: Ecol Evol. 2026 Apr 29;16(5):e73584. doi: 10.1002/ece3.73584 (PMC13127236; doi:10.1002/ece3.73584)
Supplement: Supplementary file 1 — Figure S1. Soil moisture over time in the main experiment. Data are presented as daily averages, and the line indicates the trend line. The plot illustrates the variation in soil moisture over the course of the experiment and remaining mostly near 30% soil moisture throughout the main experiment. Figure S2. A. Soil moisture over time in the drought experiment. Data are presented as daily averages, and the line indicates the trend line highlighting the differences in moisture availability between control (mean = 30.5%) and drought (mean = 15.2%) treatments after start of drought until the end of the experiment. Figure S3. Regressions from the main experiment mid‐experiment. (A) Leaf width, (B) number of leaves, (C) stem diameter, (D) stomatal conductance, (E) transpiration rate, (F) water use efficiency, (G) internal carbon dioxide concentration, (H) water potential, (I) date of bolting, (J) relative height growth rate and (K) relative leaf area growth rate. The solid line is the regression, shaded area indicates the confidence interval, points indicate site mean, and error bars indicate site standard error. The equation of the line of best fit is included. Figure S4. Regressions from the drought experiment. (A) Leaf area, (B) number of leaves, (C) stem diameter, (D) leaf thickness, (E) leaf width, (F) vegetative biomass, (G) reproductive biomass, (H) seed biomass, (I) rhizome biomass, (J) root biomass, (K) root to shoot ratio, (L) stomatal conductance, (M) transpiration rate, (N) water‐use efficiency, O. carbon dioxide concentration, (P) chlorophyll absorbance, (Q) date of bolting, (R) height‐based growth rate, and (S) leaf area‐based growth rate across a precipitation gradient. Blue indicates control and red indicates drought. The solid line is the regression, shaded area indicates the confidence interval, points indicate site mean, and error bars indicate site standard error. The equation of the line of best fit for each treatment (control or drought) is included. [file ECE3-16-e73584-s001.docx]

**Supplemental Figures**

**
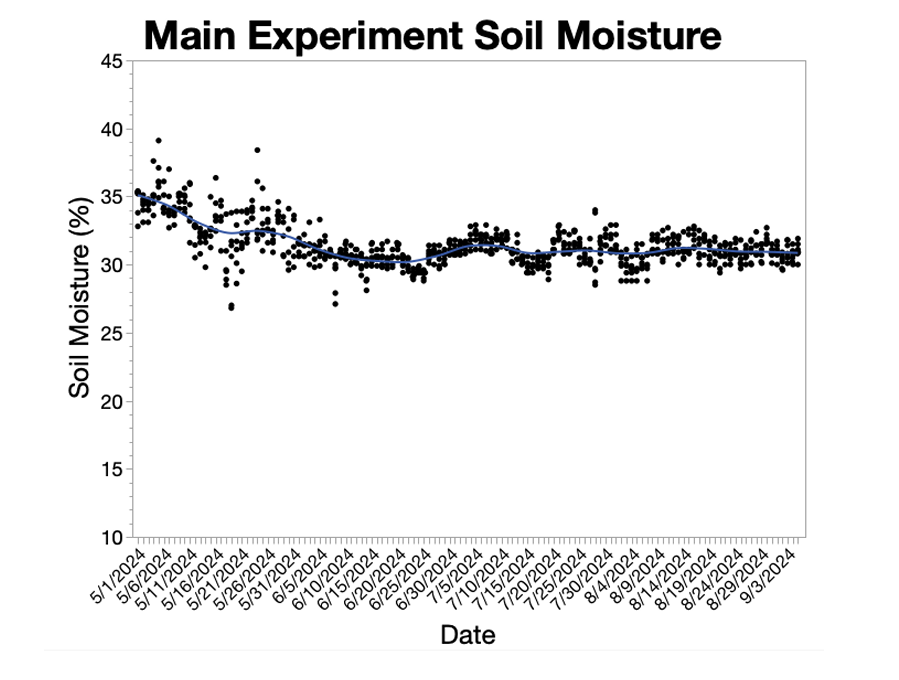
**

**Supplemental Figure 1.** Soil moisture over time in the main experiment. Data are presented as daily averages, and the line indicates the trend line. The plot illustrates the variation in soil moisture over the course of the experiment and remaining mostly near 30% soil moisture throughout the main experiment.

**
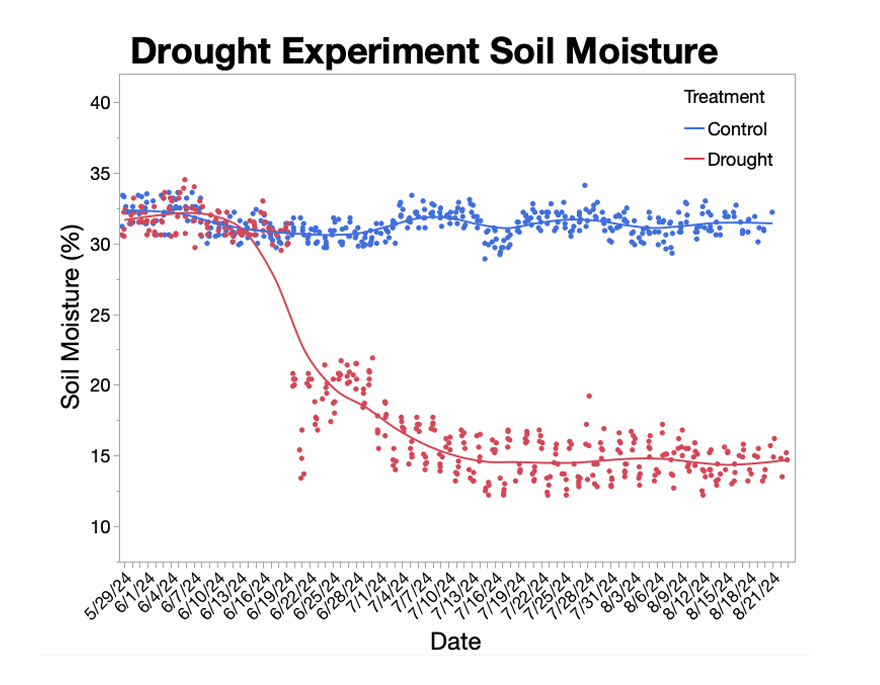
**

**Supplemental Figure 2.** A. Soil moisture over time in the drought experiment. Data are presented as daily averages, and the line indicates the trend line highlighting the differences in moisture availability between control (mean= 30.5%) and drought (mean = 15.2%) treatments after start of drought until the end of the experiment.


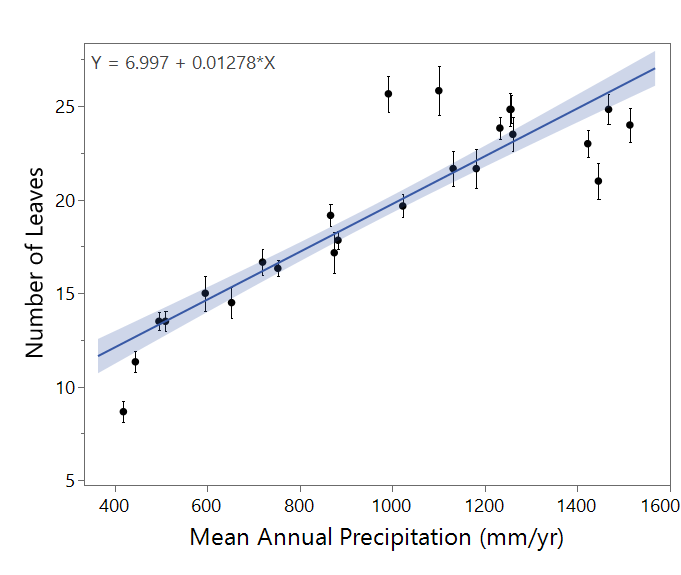

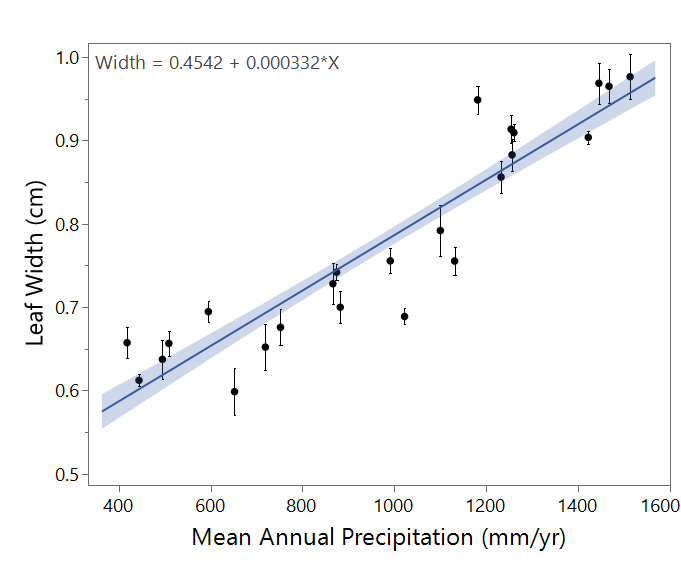

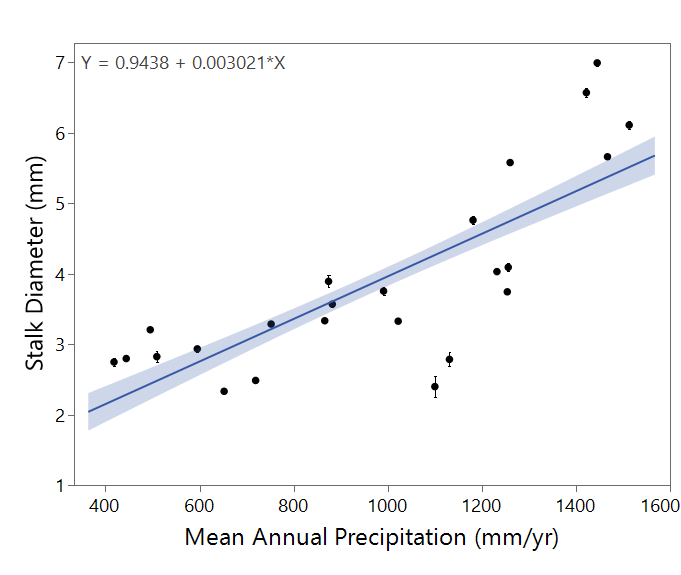

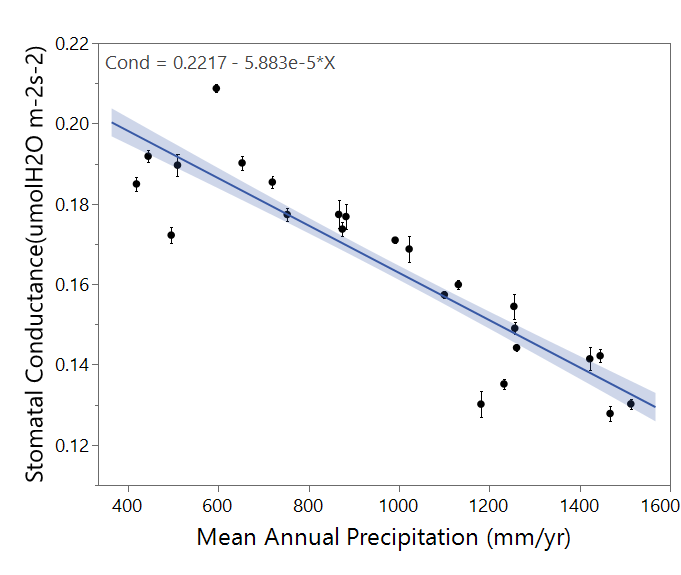


**D.**

**C.**

**B.**

**A.**


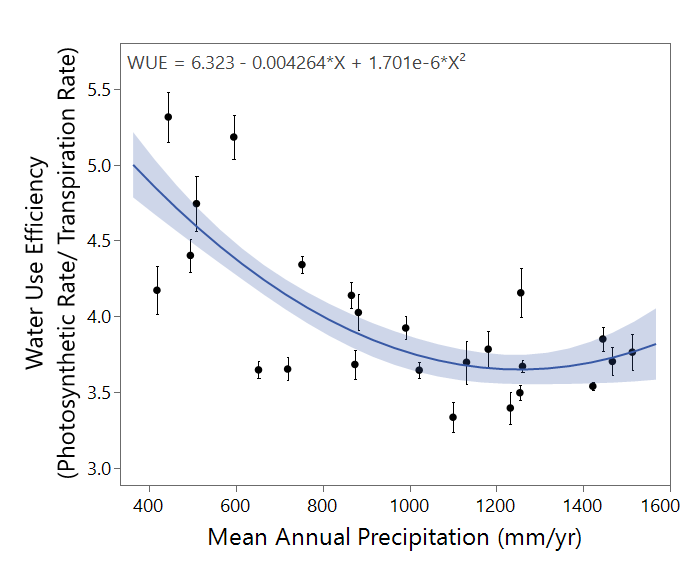

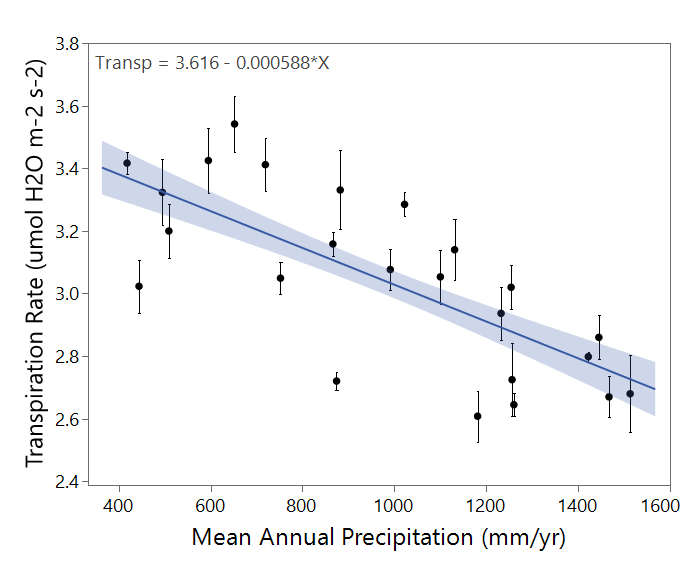

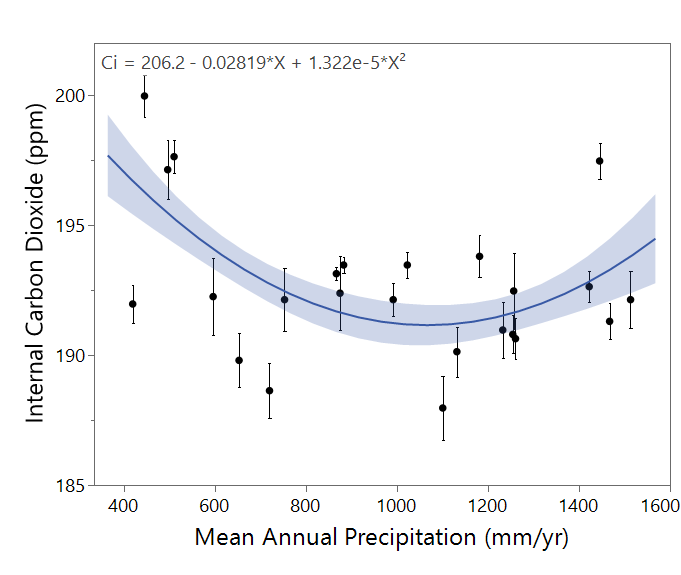

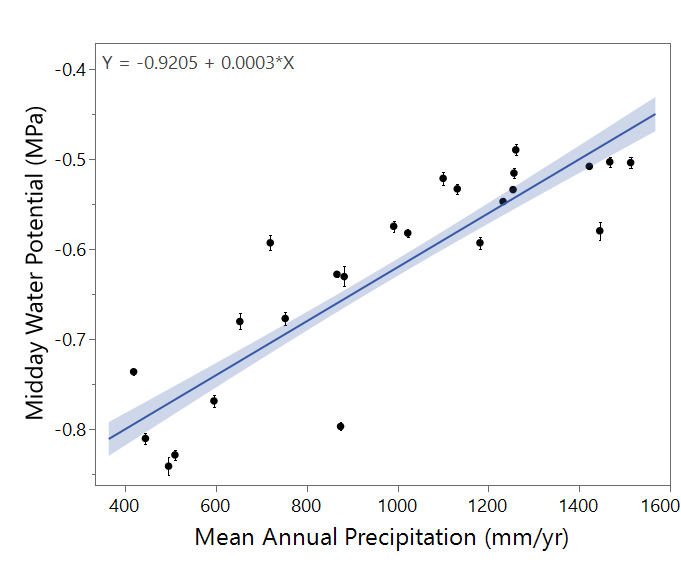


**H.**

**G.**

**F.**

**E.**


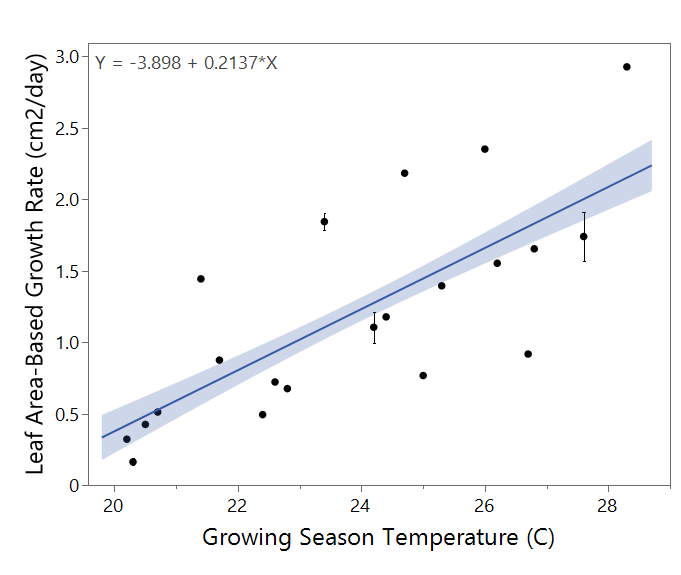

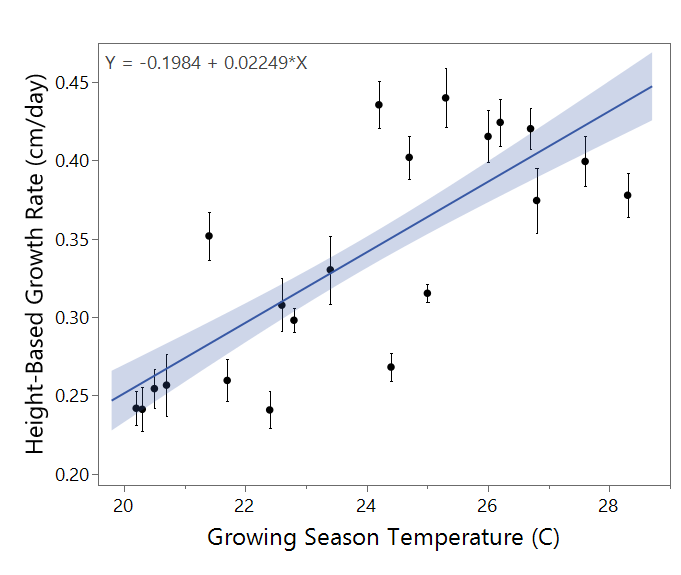

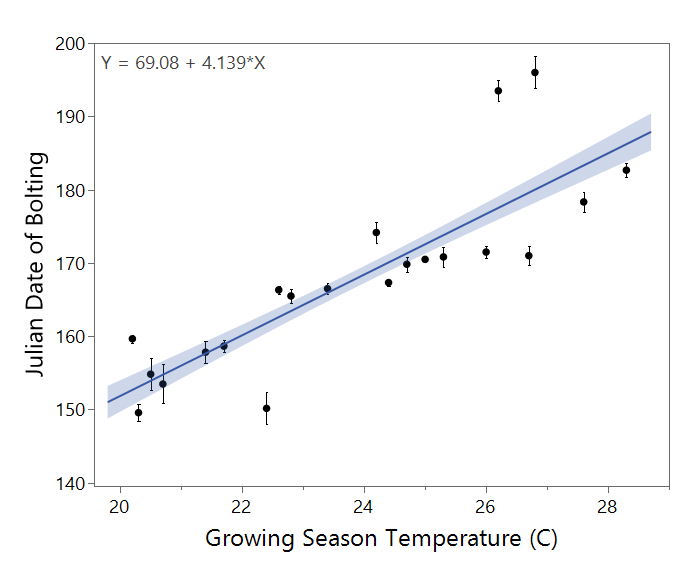


**Supplemental Figure 3.** Regressions from the main experiment mid-experiment. A. Leaf width, B. number of leaves, C. stem diameter, D. stomatal conductance, E. transpiration rate, F. water use efficiency, G. internal carbon dioxide concentration, H. water potential., I. date of bolting, J. relative height growth rate and K. relative leaf area growth rate. The solid line is the regression, shaded area indicates the confidence interval, points indicate site mean, and error bars indicate site standard error. The equation of the line of best fit is included.

**K.**

**I.**

**J.**


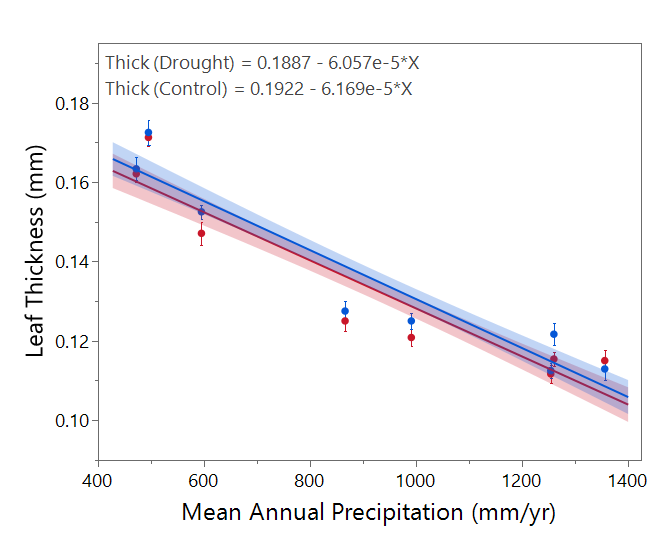

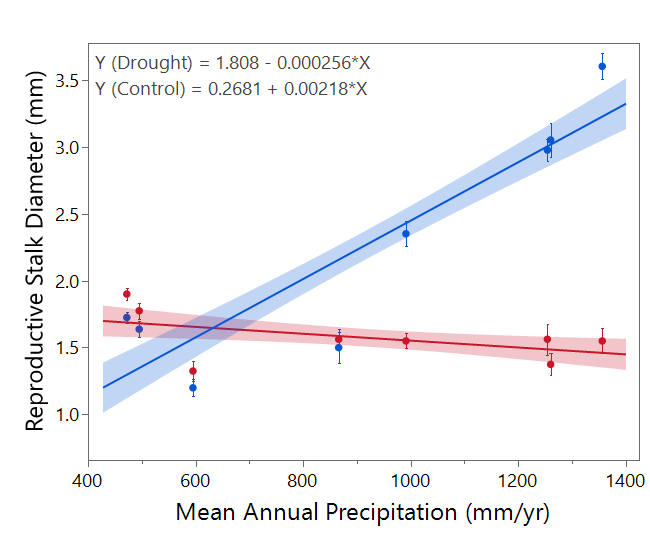

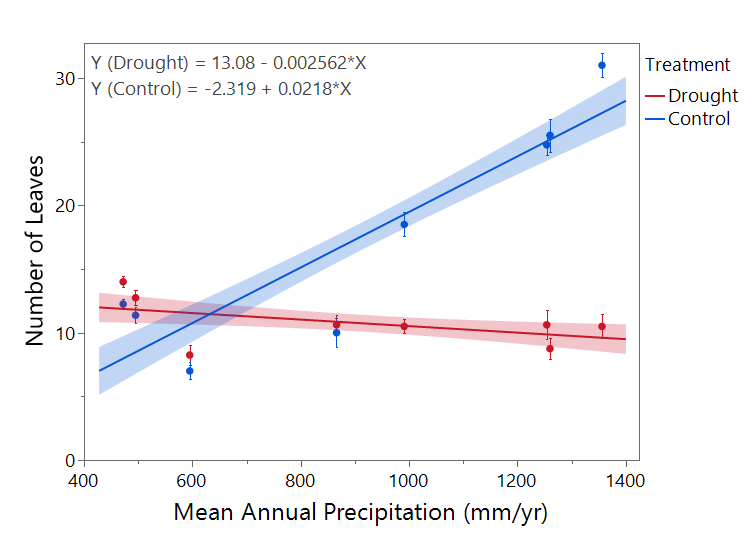

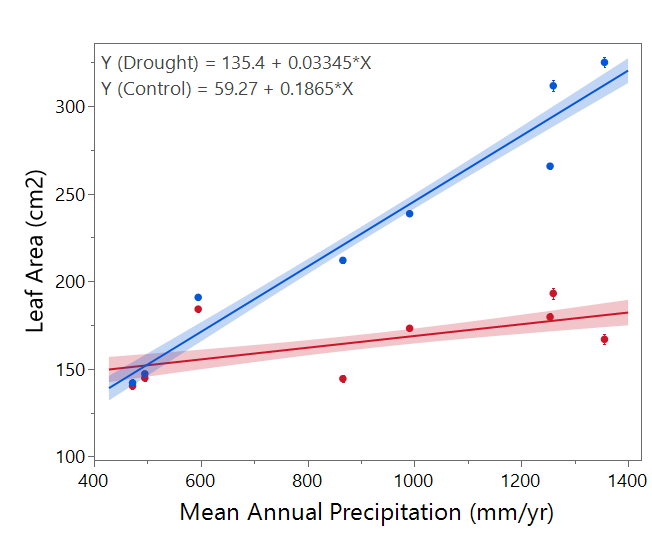


**D.**

**C.**

**B.**

**A.**


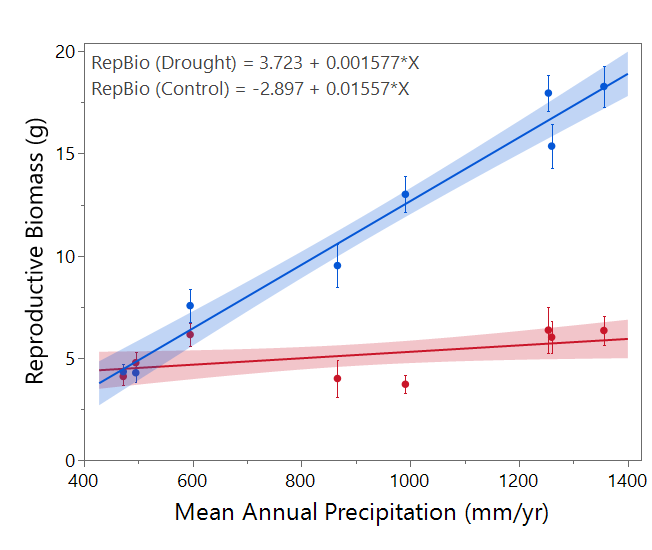

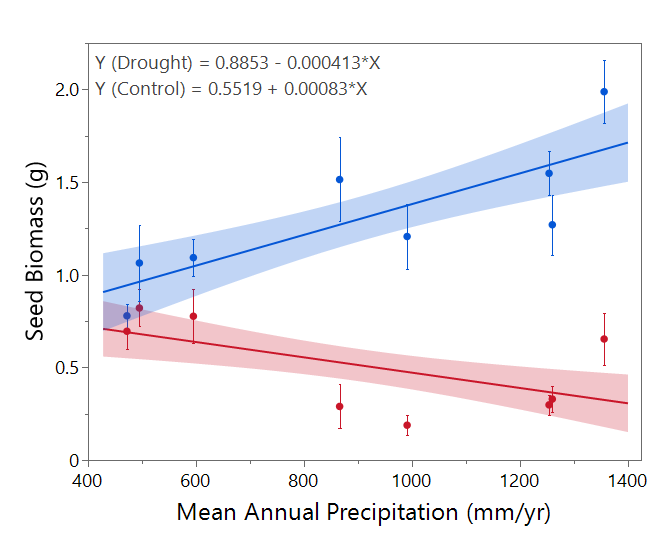

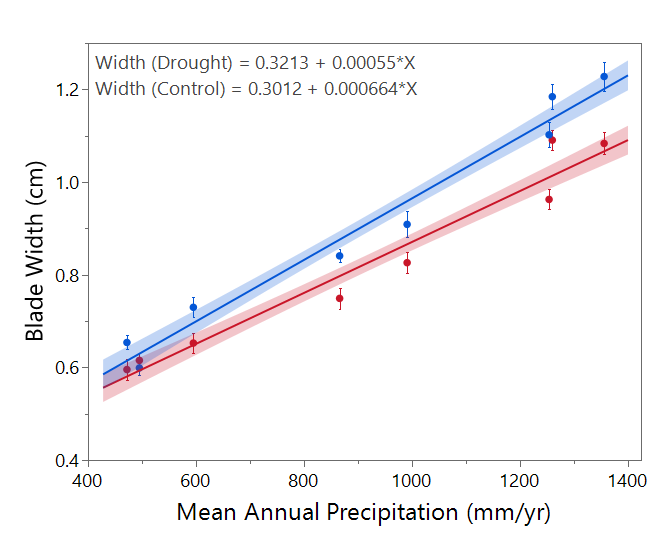

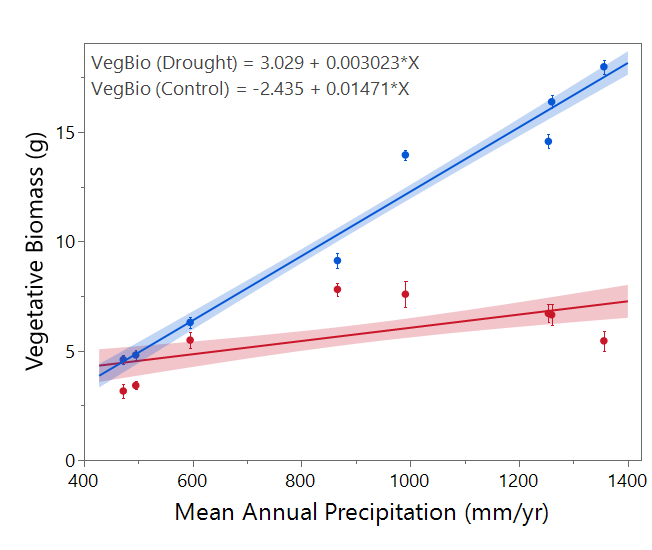


**H.**

**G.**

**F.**

**E.**


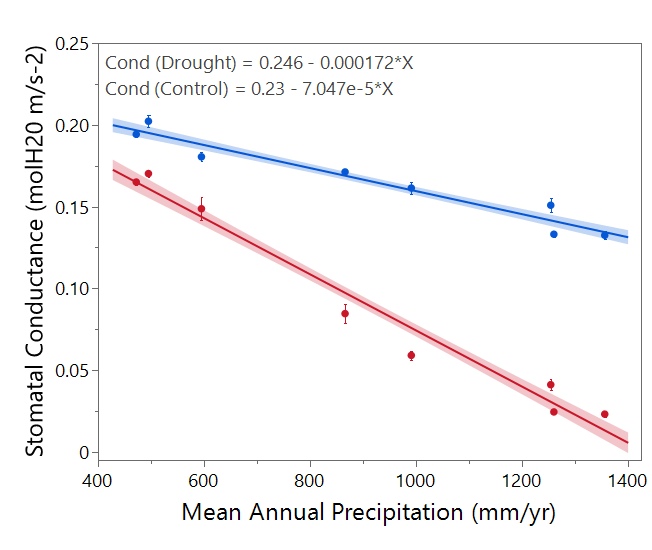

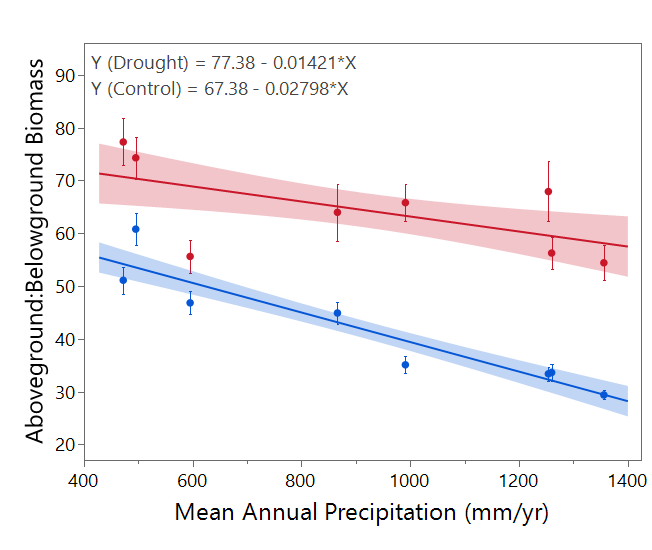

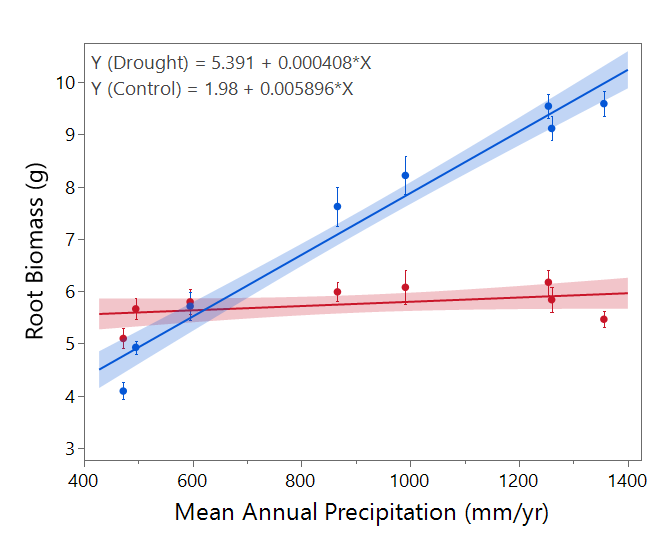

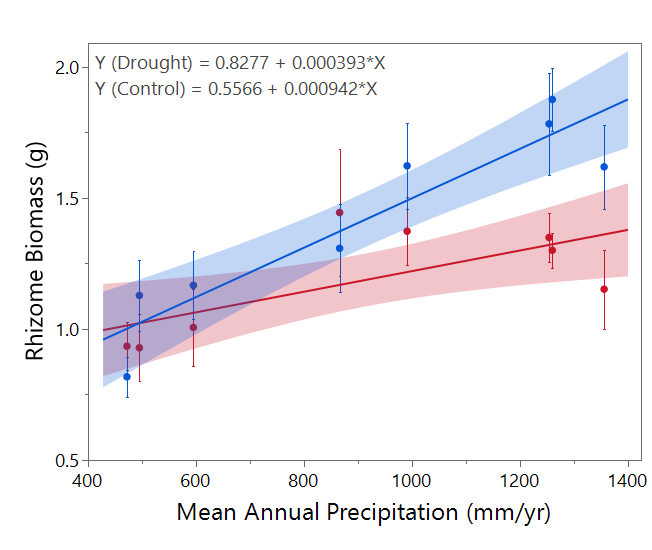


**L.**

**K.**

**I.**

**J.**


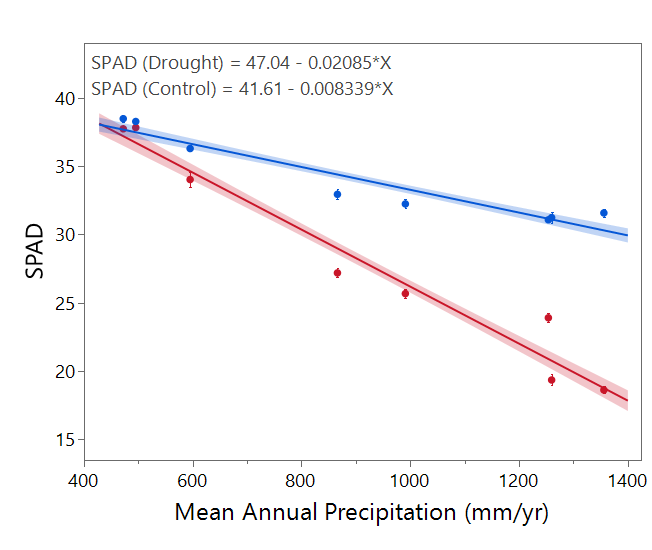

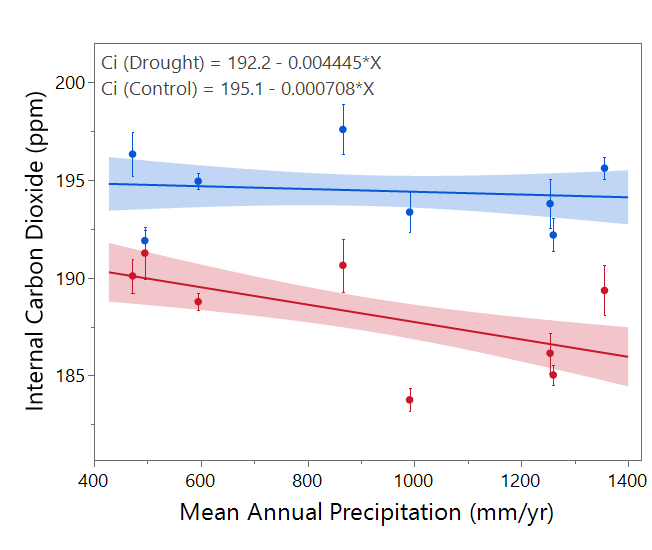

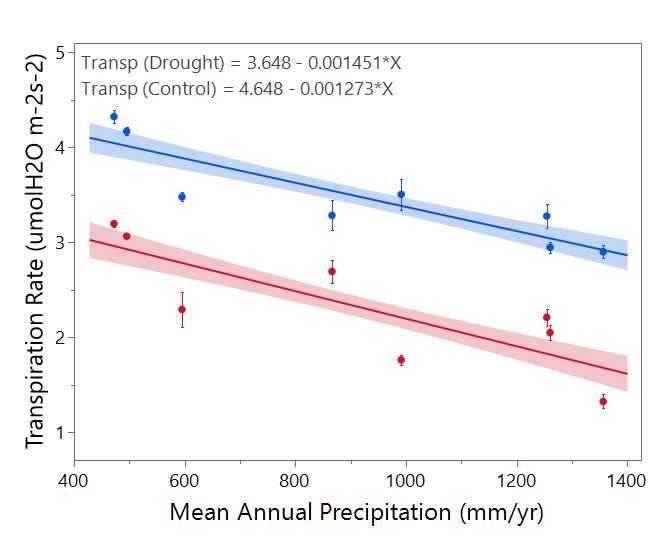

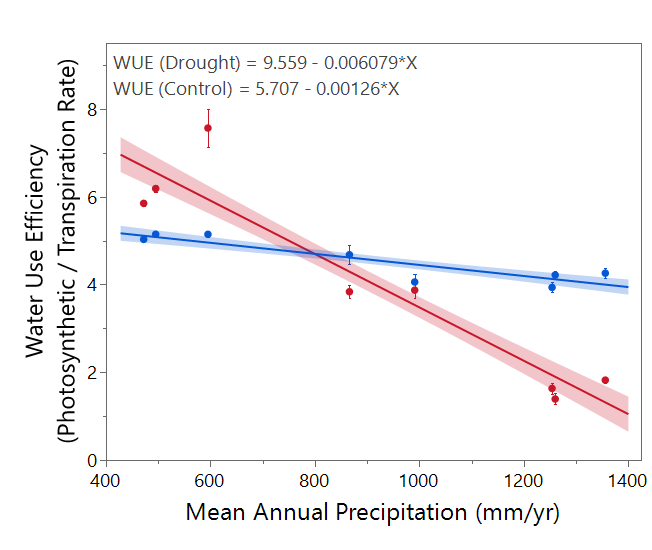


**O.**

**M.**

**P.**

**N.**


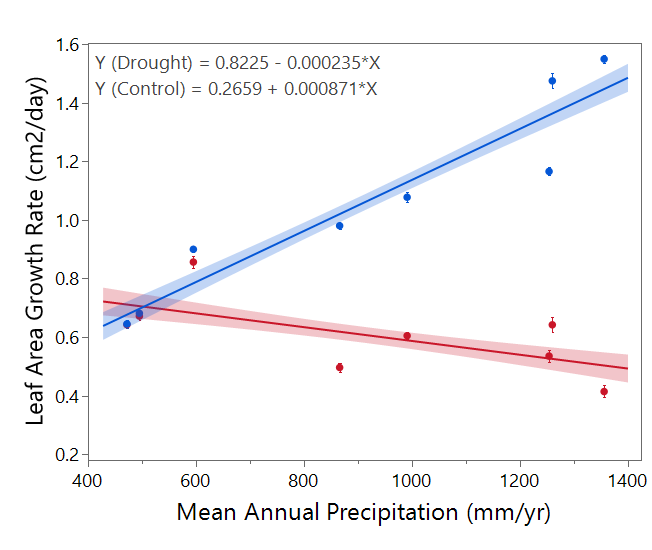

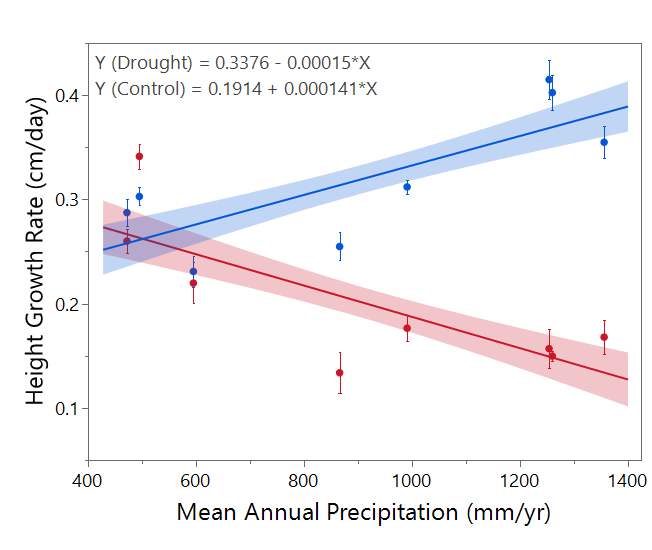

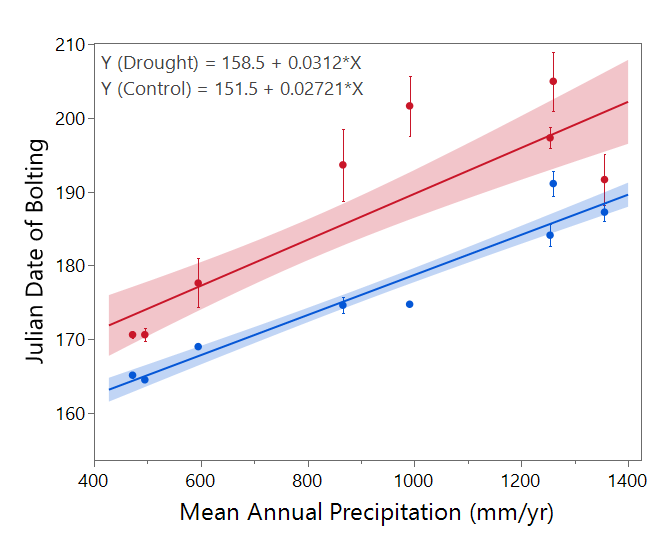


**Supplemental Figure 4.** Regressions from the drought experiment. A. Leaf area, B. number of leaves, C. stem diameter, D. leaf thickness, E. leaf width, F. vegetative biomass, G. reproductive biomass, H. seed biomass, I. rhizome biomass, J. root biomass, K. root to shoot ratio, L. stomatal conductance, M. transpiration rate, N. water-use efficiency, O. carbon dioxide concentration, P. chlorophyll absorbance, Q. date of bolting, R. height-based growth rate, and S. leaf area-based growth rate across a precipitation gradient. Blue indicates control and red indicates drought. The solid line is the regression, shaded area indicates the confidence interval, points indicate site mean, and error bars indicate site standard error. The equation of the line of best fit for each treatment (control or drought) is included.

**S.**

**R.**

**Q.**

| **Site/ State** | **County** | **Longitude** | **Latitude** | **Mean Annual Precipitation (mm)** | **Growing Season Precipitation (mm)** | **Mean Annual Temperature (C)** | **Warmest Month Temperature**  **(C)** | **Growing Season Temperature**  **(C)** | **Coldest Month Temperature (C)** |
| --- | --- | --- | --- | --- | --- | --- | --- | --- | --- |
| **AL** | Dallas | -87.12 | 32.31 | 1422 | 508 | 17.7 | 27.6 | 27.3 | 7.0 |
| **AR** | Lonoke | -91.70 | 34.77 | 1232 | 463 | 16.5 | 27.5 | 21.5 | 4.3 |
| **CO-1** | Boulder | -105.28 | 39.99 | 495 | 139 | 10.8 | 23.2 | 22.6 | 0.5 |
| **CO-2** | Boulder | -105.11 | 39.58 | 467 | 143 | 10.7 | 23.0 | 22.5 | 0.4 |
| **IA** | Jasper | -93.01 | 41.56 | 1022 | 418 | 9.7 | 23.6 | 24.7 | -6.2 |
| **IL** | Williamson | -88.83 | 38.78 | 1254 | 483 | 13.6 | 25.6 | 21.9 | 0.4 |
| **IN** | Lake | -87.45 | 41.52 | 1100 | 400 | 10.5 | 23.5 | 25.2 | -3.8 |
| **KS-1** | Riley | -96.61 | 39.22 | 866 | 325 | 12.6 | 26.1 | 24.6 | -1.7 |
| **KS-2** | Logan | -100.81 | 38.77 | 595 | 228 | 11.9 | 25.8 | 27.6 | -1.2 |
| **LA** | Ouachita Parish | -92.04 | 32.60 | 1467 | 574 | 18.5 | 28.2 | 28.8 | 7.9 |
| **MI** | Kalamazoo | -85.76 | 42.17 | 1131 | 382 | 9.6 | 22.4 | 20.9 | -4.2 |
| **MN** | Clay | -96.14 | 47.10 | 719 | 364 | 5.9 | 21.4 | 21.2 | -8.2 |
| **MO** | Calloway | -91.99 | 38.94 | 991 | 389 | 13 | 25.3 | 24.6 | -0.2 |
| **MS** | Yalobusha | -89.75 | 33.94 | 1513 | 598 | 16.7 | 26.9 | 26.8 | 5.5 |
| **MT** | Rosebud | -105.41 | 45.260 | 418 | 194 | 7.7 | 22.1 | 20.5 | -4.9 |
| **NC** | Cabarrus | -80.51 | 35.44 | 1260 | 497 | 15.6 | 26.3 | 24.8 | 4.9 |
| **ND** | Ransom | -97.30 | 46.39 | 652 | 297 | 5.8 | 21.7 | 21.2 | 2.4 |
| **NE-1** | Lancaster | -96.80 | 40.86 | 882 | 361 | 10.6 | 24.5 | 23.5 | -4.7 |
| **NE-2** | Knox | -98.06 | 42.75 | 652 | 270 | 9.5 | 24.2 | 23.3 | -5.1 |
| **NM** | Union | -104.22 | 36.23 | 444 | 170 | 11.7 | 23.5 | 22.2 | 1.0 |
| **OK** | Beckham | -99.68 | 35.61 | 509 | 240 | 7.7 | 22.4 | 20.8 | 1.2 |
| **SC** | Chesterfield | -80.13 | 34.37 | 1256 | 352 | 16.8 | 26.9 | 25.6 | 5.2 |
| **SD** | Lawrence | -103.07 | 45.10 | 654 | 184 | 14.9 | 27.2 | 26.2 | -2.6 |
| **TX-1** | Fannin | -95.03 | 29.37 | 1181 | 419 | 17.5 | 28.4 | 28.5 | 6.1 |
| **TX-2** | Galveston | -97.34 | 31.06 | 1445 | 569 | 21.2 | 28.7 | 29.5 | 7.3 |
| **TX-3** | Bell | -95.97 | 33.72 | 874 | 384 | 8.6 | 22.3 | 21.2 | 6.6 |

**Supplemental Table 1A.** Site information, location (longitude and latitude), and home climate variables showing historic annual temperature and precipitation and other relevant site details.

**Supplemental Tables**

| **Site** | **Soil Moisture (%)** | **Soil Temperature (C)** | **Soil pH** | **Soil Sand (%)** | **Soil Silt (%)** | **Soil Clay (%)** |
| --- | --- | --- | --- | --- | --- | --- |
| AL | 23.58333 | 24.17593 | 58 | 54 | 29 | 17 |
| AR | 30.35 | 22.39815 | 59 | 36 | 54 | 10 |
| CO | 14.21667 | 18.69444 | 68 | 59 | 26 | 15 |
| IA | 28.41667 | 18.37037 | 64 | 44 | 41 | 15 |
| IL | 34.5 | 22.35185 | 59 | 15 | 67 | 18 |
| IN | 33.95 | 17.0463 | 60 | 59 | 29 | 10 |
| KS-1 | 29.1 | 20.24074 | 67 | 17 | 61 | 22 |
| KS-2 | 21.16667 | 19.43519 | 72 | 43 | 40 | 17 |
| LA | 39.35 | 26.10185 | 54 | 14 | 73 | 13 |
| MI | 28.5 | 18.14815 | 64 | 67 | 19 | 14 |
| MN | 24.31667 | 17.19444 | 73 | 31 | 52 | 17 |
| MO | 23.46667 | 21.21296 | 67 | 22 | 59 | 19 |
| MS | 23.18333 | 23.24074 | 54 | 48 | 46 | 6 |
| MT | 14.65 | 18.67593 | 69 | 32 | 50 | 18 |
| NC | 29.83333 | 21.73148 | 53 | 22 | 28 | 50 |
| ND | 24.63333 | 19.37037 | 69 | 64 | 27 | 12 |
| NE-1 | 25.9 | 22.25926 | 61 | 22 | 59 | 19 |
| NE-2 | 21.91667 | 24.42593 | 71 | 20 | 32 | 48 |
| NM | 9.75 | 24.37037 | 74 | 88 | 6 | 6 |
| OK | 22.21667 | 20.99074 | 73 | 16 | 64 | 20 |
| SC | 18.71667 | 25.2963 | 65 | 26 | 59 | 15 |
| SD | 22.3 | 19.15741 | 69 | 50 | 35 | 15 |
| TX-1 | 40.65 | 24.34259 | 60 | 60 | 36 | 4 |
| TX-2 | 28.83333 | 26.58333 | 62 | 11 | 37 | 51 |
| TX-3 | 13.66667 | 25.46296 | 76 | 22 | 38 | 40 |

**Supplemental Table 1B.** Site information with home site soil moisture, temperature, pH, and texture.


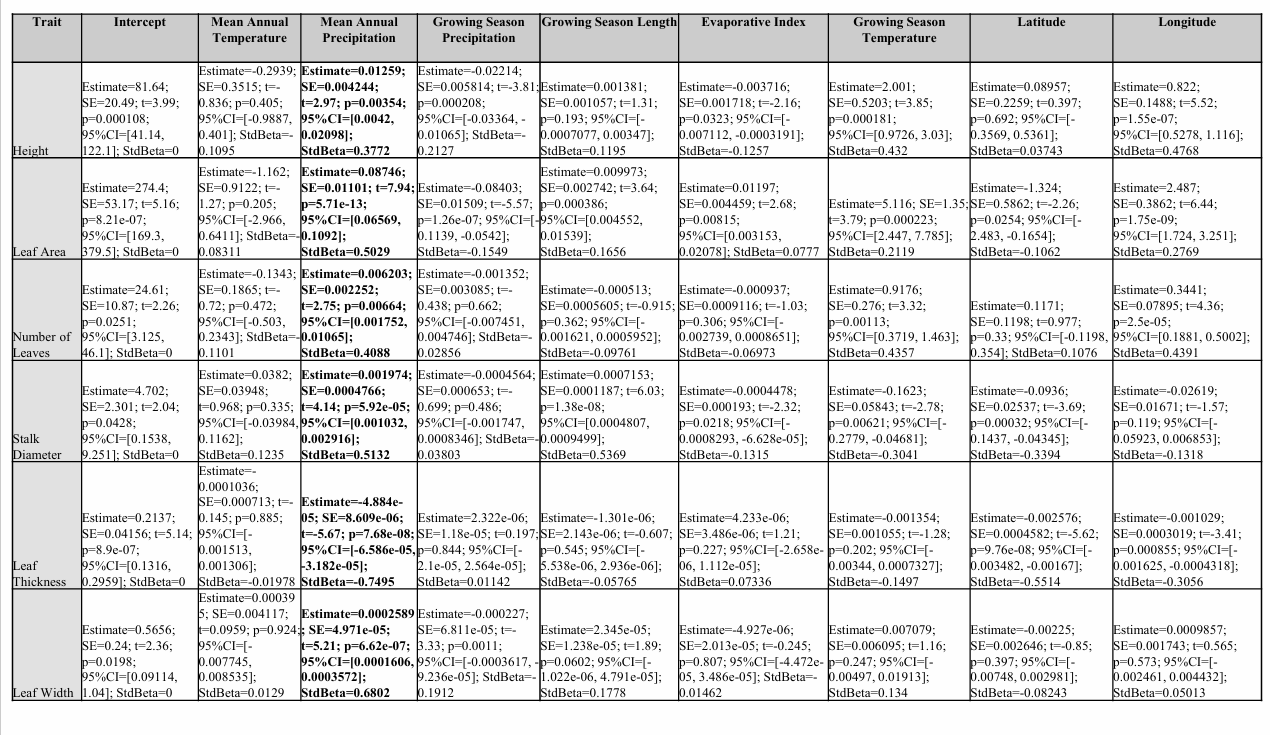


**Supplemental Table 2A.** The results of regression analysis performed to analyze the effects of home on morphological trait variation in the main experiment. The most explanatory climate predictor is in bold for each trait.


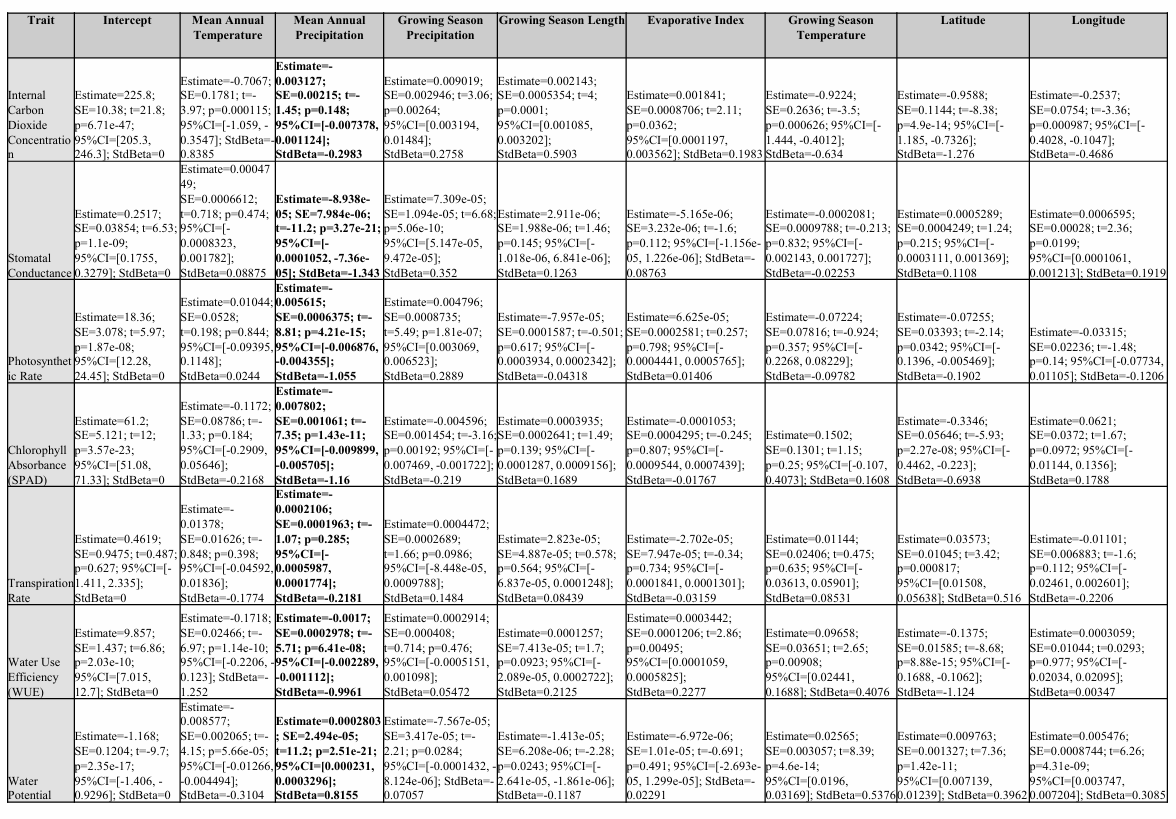


**Supplemental Table 2B.** The results of regression analysis performed to analyze the effects of home on physiological trait variation in the main experiment. The most explanatory climate predictor is in bold for each trait.


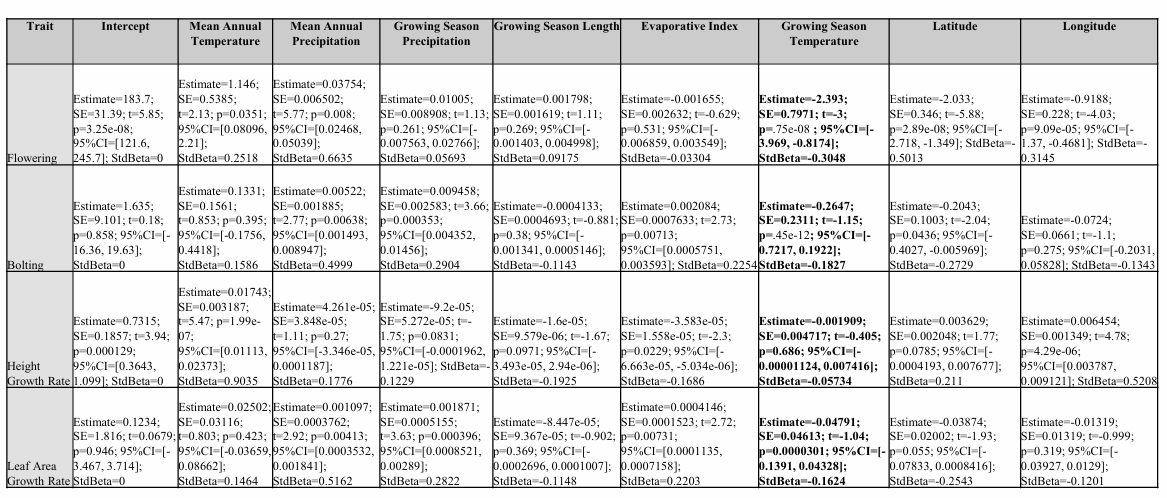


**Supplemental Table 2C.** The results of regression analysis performed to analyze the effects of home on phenological and growth-related trait variation in the main experiment. The most explanatory climate predictor is in bold for each trait.

| Trait | Mean Annual Temperature | Mean Annual Precipitation | Growing Season Precipitation | Growing Season Length | Evaporative Index | Growing Season Temperature | Latitude | Longitude |
| --- | --- | --- | --- | --- | --- | --- | --- | --- |
| Internal Carbon Dioxide Concentration | R²m = 0.38; R²c = 0.46 | **R²m = 0.62; R²c = 0.70** | R²m = 0.41; R²c = 0.49 | R²m = 0.35; R²c = 0.43 | R²m = 0.32; R²c = 0.40 | R²m = 0.44; R²c = 0.52 | R²m = 0.47; R²c = 0.55 | R²m = 0.36; R²c = 0.44 |
| Stomatal Conductance | R²m = 0.52; R²c = 0.60 | **R²m = 0.84; R²c = 0.92** | R²m = 0.58; R²c = 0.66 | R²m = 0.33; R²c = 0.41 | R²m = 0.29; R²c = 0.37 | R²m = 0.36; R²c = 0.44 | R²m = 0.31; R²c = 0.39 | R²m = 0.42; R²c = 0.50 |
| Photosynthetic Rate | R²m = 0.34; R²c = 0.42 | **R²m = 0.56; R²c = 0.64** | R²m = 0.39; R²c = 0.47 | R²m = 0.11; R²c = 0.19 | R²m = 0.09; R²c = 0.17 | R²m = 0.18; R²c = 0.26 | R²m = 0.23; R²c = 0.31 | R²m = 0.14; R²c = 0.22 |
| Chlorophyll Absorbance (SPAD) | R²m = 0.22; R²c = 0.30 | **R²m = 0.66; R²c = 0.74** | R²m = 0.28; R²c = 0.36 | R²m = 0.16; R²c = 0.24 | R²m = 0.08; R²c = 0.16 | R²m = 0.17; R²c = 0.25 | R²m = 0.49; R²c = 0.57 | R²m = 0.13; R²c = 0.21 |
| Transpiration Rate | R²m = 0.19; R²c = 0.27 | **R²m = 0.48; R²c = 0.56** | R²m = 0.21; R²c = 0.29 | R²m = 0.10; R²c = 0.18 | R²m = 0.07; R²c = 0.15 | R²m = 0.15; R²c = 0.23 | R²m = 0.41; R²c = 0.49 | R²m = 0.12; R²c = 0.20 |
| Water Use Efficiency (WUE) | R²m = 0.43; R²c = 0.51 | **R²m = 0.67; R²c = 0.75** | R²m = 0.16; R²c = 0.24 | R²m = 0.18; R²c = 0.26 | R²m = 0.24; R²c = 0.32 | R²m = 0.31; R²c = 0.39 | R²m = 0.52; R²c = 0.60 | R²m = 0.05; R²c = 0.13 |
| Water Potential | R²m = 0.46; R²c = 0.54 | **R²m = 0.71; R²c = 0.79** | R²m = 0.29; R²c = 0.37 | R²m = 0.21; R²c = 0.29 | R²m = 0.19; R²c = 0.27 | R²m = 0.33; R²c = 0.41 | R²m = 0.57; R²c = 0.65 | R²m = 0.39; R²c = 0.47 |

**Supplemental Table 2D.** The results of regression analysis performed to analyze the effects of home on physiological, morphological, and phenological traits reporting the variance explained by R^2^m and R^2^c.

| Trait | Mean Annual Temperature | Mean Annual Precipitation | Growing Season Precipitation | Growing Season Length | Evaporative Index | Growing Season Temperature | Latitude | Longitude |
| --- | --- | --- | --- | --- | --- | --- | --- | --- |
| Height | R²m = 0.50; R²c = 0.58 | **R²m = 0.85; R²c = 0.93** | R²m = 0.71; R²c = 0.79 | R²m = 0.32; R²c = 0.40 | R²m = 0.28; R²c = 0.36 | R²m = 0.44; R²c = 0.52 | R²m = 0.47; R²c = 0.55 | R²m = 0.35; R²c = 0.43 |
| Leaf Area | R²m = 0.62; R²c = 0.70 | **R²m = 0.79; R²c = 0.87** | R²m = 0.73; R²c = 0.81 | R²m = 0.30; R²c = 0.38 | R²m = 0.26; R²c = 0.34 | R²m = 0.53; R²c = 0.61 | R²m = 0.48; R²c = 0.56 | R²m = 0.37; R²c = 0.45 |
| Number of Leaves | R²m = 0.74; R²c = 0.82 | **R²m = 0.71; R²c = 0.79** | R²m = 0.83; R²c = 0.91 | R²m = 0.34; R²c = 0.42 | R²m = 0.29; R²c = 0.37 | R²m = 0.49; R²c = 0.57 | R²m = 0.46; R²c = 0.54 | R²m = 0.33; R²c = 0.41 |
| Stalk Diameter | R²m = 0.83; R²c = 0.91 | **R²m = 0.62; R²c = 0.70** | R²m = 0.79; R²c = 0.87 | R²m = 0.31; R²c = 0.39 | R²m = 0.27; R²c = 0.35 | R²m = 0.45; R²c = 0.53 | R²m = 0.42; R²c = 0.50 | R²m = 0.36; R²c = 0.44 |
| Leaf Thickness | R²m = 0.50; R²c = 0.58 | **R²m = 0.62; R²c = 0.70** | R²m = 0.65; R²c = 0.73 | R²m = 0.28; R²c = 0.36 | R²m = 0.25; R²c = 0.33 | R²m = 0.40; R²c = 0.48 | R²m = 0.44; R²c = 0.52 | R²m = 0.32; R²c = 0.40 |
| Leaf Width | R²m = 0.81; R²c = 0.89 | **R²m = 0.49; R²c = 0.57** | R²m = 0.71; R²c = 0.79 | R²m = 0.29; R²c = 0.37 | R²m = 0.24; R²c = 0.32 | R²m = 0.46; R²c = 0.54 | R²m = 0.41; R²c = 0.49 | R²m = 0.35; R²c = 0.43 |
| Flowering | **R²m = 0.70; R²c = 0.78** | R²m = 0.42; R²c = 0.50 | R²m = 0.39; R²c = 0.47 | R²m = 0.30; R²c = 0.38 | R²m = 0.27; R²c = 0.35 | R²m = 0.65; R²c = 0.73 | R²m = 0.33; R²c = 0.41 | R²m = 0.28; R²c = 0.36 |
| Bolting | **R²m = 0.68; R²c = 0.76** | R²m = 0.40; R²c = 0.48 | R²m = 0.38; R²c = 0.46 | R²m = 0.28; R²c = 0.36 | R²m = 0.25; R²c = 0.33 | R²m = 0.63; R²c = 0.71 | R²m = 0.31; R²c = 0.39 | R²m = 0.27; R²c = 0.35 |
| Height Growth Rate | **R²m = 0.44; R²c = 0.52** | R²m = 0.28; R²c = 0.36 | R²m = 0.25; R²c = 0.33 | R²m = 0.22; R²c = 0.30 | R²m = 0.20; R²c = 0.28 | R²m = 0.41; R²c = 0.49 | R²m = 0.26; R²c = 0.34 | R²m = 0.21; R²c = 0.29 |
| Leaf Area Growth Rate | **R²m = 0.53; R²c = 0.61** | R²m = 0.31; R²c = 0.39 | R²m = 0.29; R²c = 0.37 | R²m = 0.24; R²c = 0.32 | R²m = 0.21; R²c = 0.29 | R²m = 0.50; R²c = 0.58 | R²m = 0.27; R²c = 0.35 | R²m = 0.22; R²c = 0.30 |

| **Response Variable**  **Supplemental Table 3A.** The results of general linear models (GLMs) performed to analyze the effects of drought treatment, population origin, and their interaction on trait variation. Significant population x treatment interaction in various time points predrought or after start of drought are in bold for each response variable. | **Estimate** | **Standard Error** | **Treatment F** | **Treatment P** | **Precipitation F** | **Precipitation P** | **Treatment × Precipitation F** | **Treatment × Precipitation P** |
| --- | --- | --- | --- | --- | --- | --- | --- | --- |
| **Rhizome Biomass** | 0.169 | 0.021 | 10.26 | **0.0017*** | 48.27 | **<0.0001*** | 8.16 | **0.0050*** |
| **Root Biomass** | 0.552 | 0.070 | 151.05 | **<0.0001*** | 335.30 | **<0.0001*** | 254.14 | **<0.0001*** |
| **Belowground Biomass** | 0.784 | 0.099 | 139.30 | **<0.0001*** | 316.30 | **<0.0001*** | 197.56 | **<0.0001*** |
| **Aboveground:Belowround Ratio** | 105.57 | 13.45 | 158.58 | **<0.0001*** | 63.67 | **<0.0001*** | 6.78 | **0.0103*** |
| **Vegetative Biomass** | 2.21 | 0.280 | 402.03 | **<0.0001*** | 720.09 | **<0.0001*** | 312.75 | **<0.0001*** |
| **Reproductive Biomass** | 5.21 | 0.667 | 230.69 | **<0.0001*** | 251.35 | **<0.0001*** | 167.39 | **<0.0001*** |
| **Seed Biomass** | 0.175 | 0.022 | 118.18 | **<0.0001*** | 3.996 | **0.0478*** | 35.60 | **<0.0001*** |
| **Stalk Diameter** | 0.127 | 0.016 | 119.96 | **<0.0001*** | 122.89 | **<0.0001*** | 197.05 | **<0.0001*** |
| **Number of Leaves** | 12.72 | 1.62 | 119.96 | **<0.0001*** | 122.89 | **<0.0001*** | 197.05 | **<0.0001*** |
| **Height** | 8.95 | 0.54 | 6.84 | **<0.0001*** | 8.80 | **<0.0001*** | 13.29 | **0.0001*** |
| **Leaf Area** | 2.34 | 0.73 | 16.14 | **<0.0001*** | 18.89 | **<0.0001*** | 16.38 | **<0.0001*** |
| **Leaf Thickness** | 0.045 | 0.0034 | 3.246 | 0.074 | 8.97 | **0.0033*** | 2.57 | 0.11 |
| **Leaf Width** | 0.41 | 0.0058 | 1.46 | 0.18 | 15.52 | **<0.0001*** | 0.0039 | 0.33 |
| **Aboveground Biomass** | 6.86 | 0.87 | 727.74 | **<0.0001*** | 788.46 | **<0.0001*** | 494.23 | **<0.0001*** |
| **Vegetative: Reproductive** | 0.62 | 0.080 | 8.97 | **0.0033*** | 2.57 | 0.1117 | 3.25 | 0.0741 |
| **Leaf Area Growth Rate** | 0011 | 0.0015 | 570.82 | **<0.0001*** | 130.92 | **<0.0001*** | 396.52 | **<0.0001*** |
| **Height Growth Rate** | 0.0032 | 0.00041 | 145.29 | **<0.0001*** | 0.10 | 0.7526 | 106.76 | **<0.0001*** |
| **Chlorophyll Absorbance** | 2.16 | 0.27 | 543.99 | **<0.0001*** | 1772.15 | **<0.0001*** | 325.61 | **<0.0001*** |
| **Photosynthetic Rate** | 1.065 | 0.14 | 109.18 | **<0.0001*** | 385.66 | **<0.0001*** | 375.56 | **<0.0001*** |
| **Transpiration Rate** | 0.1085 | 0.014 | 121.97 | **<0.0001*** | 10.45 | **0.0016*** | 200.51 | **0.021*** |
| **Stomatal Conductance** | 0.00015 | 0.000019 | 279.04 | **<0.0001*** | 954.40 | **<0.0001*** | 341.51 | **<0.0001*** |
| **Water Use Efficiency** | 0.49 | 0.062 | 19.54 | **<0.0001*** | 464.96 | **<0.0001*** | 200.51 | **<0.0001*** |
| **Water Potential** | 0.0061 | 0.00077 | 169.68 | **<0.0001*** | 1276.17 | **<0.0001*** | 4.16 | **0.0435*** |
| **Bolting** | 41.79 | 5.79 | 46.02 | **<0.0001*** | 203.22 | **<0.0001*** | 0.95 | 0.3331 |
| **Flowering** | 31.80 | 4.41 | 76.42 | **<0.0001*** | 203.22 | **<0.0001*** | 2.99 | **0.0034*** |

| **Response Variable** | **Population** | **Home Precip** | **Before D1** | **Before D2** | **Drought T1** | **Drought T2** | **Drought T3** | **Drought T4** | **Drought T5** | **Drought T6** | **Drought T7** | **Drought T8** | **F (df = 15)** | **Marginal R²** |
| --- | --- | --- | --- | --- | --- | --- | --- | --- | --- | --- | --- | --- | --- | --- |
| Vegetative height (cm) | <0.001 | <0.001 | 0.59 | 0.82 | 0.006 | <0.001 | <0.001 | <0.001 | <0.001 | <0.001 | <0.001 | <0.001 | 18.94 | 0.94 |
| Blade width (cm) | <0.001 | <0.001 | 0.99 | — | 0.04 | 0.083 | — | — | — | — | — | — | 2.11 | 0.11 |
| Leaf area (cm²) | <0.001 | <0.001 | 0.82 | — | <0.001 | <0.001 | — | — | — | — | — | — | 9.87 | 0.33 |
| Leaf thickness (mm) | <0.001 | <0.001 | 0.58 | — | 0.73 | 0.98 | — | — | — | — | — | — | 1.02 | 0.09 |
| Number of leaves | <0.001 | <0.001 | 0.66 | — | 0.02 | — | — | — | — | — | — | — | 8.41 | 0.75 |
| Stalk diameter (mm) | <0.001 | <0.001 | — | — | 0.01 | — | — | — | — | — | — | — | 8.16 | 0.75 |

**Supplemental Table 4A.** The results of general linear models (GLMs) of morphological traits performed to analyze the effects of drought treatment, population origin, and their interaction on trait variation over time (e.g., Drought T2 = the effect of drought treatment on time period = 2).

| **Response Variable** | **Population** | **Home Precip** | **Before D1** | **Before D2** | **Drought T1** | **Drought T2** | **Drought T3** | **Drought T4** | **Drought T5** | **Drought T6** | **Drought T7** | **Drought T8** | **F (df = 15)** | **Marginal R²** |
| --- | --- | --- | --- | --- | --- | --- | --- | --- | --- | --- | --- | --- | --- | --- |
| Chlorophyll absorbance (SPAD) | <0.001 | <0.001 | 0.61 | 0.63 | <0.001 | <0.001 | <0.001 | <0.001 | <0.001 | <0.001 | <0.001 | <0.001 | 14.72 | 0.86 |
| Photosynthetic rate | <0.001 | <0.001 | 0.47 | — | <0.001 | <0.001 | <0.001 | <0.001 | — | — | — | — | 17.32 | 0.91 |
| Transpiration rate | <0.001 | <0.001 | 0.73 | — | 0.003 | 0.002 | 0.04 | <0.001 | — | — | — | — | 9.41 | 0.41 |
| Stomatal conductance | <0.001 | <0.001 | 0.33 | — | <0.001 | 0.01 | <0.001 | <0.001 | — | — | — | — | 15.08 | 0.86 |
| Water-use efficiency | <0.001 | <0.001 | 0.88 | — | <0.001 | <0.001 | <0.001 | <0.001 | — | — | — | — | 11.64 | 0.55 |
| Water potential (MPa) | <0.001 | <0.001 | 0.59 | — | 0.03 | <0.001 | 0.03 | — | — | — | — | — | 6.82 | 0.36 |
| Internal CO₂ | <0.001 | <0.001 | 0.20 | — | <0.001 | 0.04 | 0.03 | 0.07 | — | — | — | — | 5.91 | 0.31 |

**Supplemental Table 4B.** The results of general linear models (GLMs) of physiological traits performed to analyze the effects of drought treatment, population origin, and their interaction on trait variation over time (e.g., Drought T2 = the effect of drought treatment on time period = 2).

| **Response Variable** | **Population** | **Home Precip** | **Drought T1** | **Drought T2** | **F (df = 15)** | **Marginal R²** |
| --- | --- | --- | --- | --- | --- | --- |
| Bolting | <0.001 | <0.001 | 0.02 | — | 6.42 | 0.58 |
| Flowering | <0.001 | <0.001 | <0.001 | — | 11.87 | 0.83 |
| Relative growth rate (height-based) | <0.001 | <0.001 | <0.001 | — | 9.64 | 0.47 |
| Relative growth rate (leaf-area–based) | <0.001 | <0.001 | <0.001 | — | 10.21 | 0.49 |

**Supplemental Table 4C.** The results of general linear models (GLMs) of phenological and growth-related traits performed to analyze the effects of drought treatment, population origin, and their interaction on trait variation over time (e.g., Drought T2 = the effect of drought treatment on time period = 2).
